# A mathematical model of hiPSC cardiomyocytes electromechanics

**DOI:** 10.14814/phy2.15124

**Published:** 2021-11-25

**Authors:** Mohamadamin Forouzandehmehr, Jussi T. Koivumäki, Jari Hyttinen, Michelangelo Paci

**Affiliations:** ^1^ Faculty of Medicine and Health Technology Tampere University Tampere Finland

**Keywords:** action potential, contractility, drug test, human stem cell‐derived cardiomyocyte, immature cardiomyocytes, in silico modeling

## Abstract

Human induced pluripotent stem cell‐derived cardiomyocytes (hiPSC‐CMs) are becoming instrumental in cardiac research, human‐based cell level cardiotoxicity tests, and developing patient‐specific care. As one of the principal functional readouts is contractility, we propose a novel electromechanical hiPSC‐CM computational model named the hiPSC‐CM‐CE. This model comprises a reparametrized version of contractile element (CE) by Rice et al., 2008, with a new passive force formulation, integrated into a hiPSC‐CM electrophysiology formalism by Paci et al. in 2020. Our simulated results were validated against *in vitro* data reported for hiPSC‐CMs at matching conditions from different labs. Specifically, key action potential (AP) and calcium transient (CaT) biomarkers simulated by the hiPSC‐CM‐CE model were within the experimental ranges. On the mechanical side, simulated cell shortening, contraction–relaxation kinetic indices (RT_50_ and RT_25_), and the amplitude of tension fell within the experimental intervals. Markedly, as an inter‐scale analysis, correct classification of the inotropic effects due to non‐cardiomyocytes in hiPSC‐CM tissues was predicted on account of the passive force expression introduced to the CE. Finally, the physiological inotropic effects caused by Verapamil and Bay‐K 8644 and the aftercontractions due to the early afterdepolarizations (EADs) were simulated and validated against experimental data. In the future, the presented model can be readily expanded to take in pharmacological trials and genetic mutations, such as those involved in hypertrophic cardiomyopathy, and study arrhythmia trigger mechanisms.

## Graphical abstract

We present the first hiPSC‐CM computational model that accounts for essential AP, CaT, and mechanical biomarkers incorporating experimental variability. The introduced passive force handling enables the model to capture the inotropic effect of non‐cardiomyocytes in hiPSC‐CM tissues. Simulated cell shortening and contraction–relaxation indices fall within experimental ranges, and EAD‐based aftercontractions predicted by the model are in accord with experimental observations. Predicted Verapamil and Bay‐K 8644 inotropic effects agree with in vitro data.

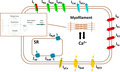

## 1 INTRODUCTION

The rapid development of engineered heart tissue approaches opens novel avenues for drug trials and investigating heart diseases, building on the recent advancements in producing hiPSC‐CMs. Furthermore, by holding the same genetic information as the donor, hiPSC‐CM‐based methods hold great promise for developing patient‐specific treatment options. As the interest in investigating the contractile indices of hiPSC‐CMs grows, *in vitro* measurements of contractility, such as video microscopy (Ahola et al., 2018), are becoming a standard alongside the more classical electrophysiological studies, such as patch‐clamp experiments. Accordingly, there is a great need for developing comprehensive computational models of hiPSC‐CMs that describe the biomechanical function, in addition to electrophysiology. Aside from the chance to better understand the underlying physiology, the more advanced models would answer the pressing need for drug effect calculations and cardiotoxicity predictions. The idea has also been advocated by the Comprehensive In Vitro Proarrhythmia Assay (CiPA) initiative (Strauss et al., 2019).

Mathematical models in cardiology, developed on different scales, have been growing in complexity, thanks to the progress in the predictive power of computer‐based frameworks (Amani et al., 2021; Forouzandehmehr & Shamloo, 2018, 2021; Hossain et al., 2014; Muller et al., 2014; Paci et al., 2013; Shamloo et al., 2019; Shamloo & Forouzandehmehr, 2019; Zile & Trayanova, 2018). The dominating focus in the mathematical modeling of cardiac or muscle cells has been the electrophysiology (Bartolucci et al., 2020; Grandi et al., 2010; Kernik et al., 2019; O’Hara et al., 2011; Paci, Pölönen, et al., 2018; Tomek et al., 2019; Tusscher & Panfilov, 2006), and the electromechanical aspect has received much less attention. In some non‐human multi‐scale mathematical models, especially for the mouse and rat, elements of myofilament contraction are at play with a detailed biophysical formalism (Rice et al., 2008; Campbell et al., 2010; Land et al., 2013; Land & Niederer, 2015; Sheikh et al., 2012). The alterations in intracellular properties can be associated with entire organ mechanical outcomes using such models. Moreover, based on modifications to channels or the proteins involved in Ca^2+^ and contractile regulations, these models were successful in the prediction of key cardiac function indices. To include the mechanical aspects of cardiomyocytes, there have been efforts to establish human biophysical models of tension production capable of integration into a whole organ contraction assembly (Land et al., 2017). Furthermore, some models of human ventricular cardiomyocyte (hV‐CM) electromechanics simulating active tensions and sarcomere dynamics (Margara et al., 2020, 2021) have also been introduced. However, there is a lack of biophysical models of whole hiPSC‐CM electromechanics, which can predict active tension, cell shortening, and particularly inotropic effect of non‐cardiomyocytes (Iseoka et al., 2018).

This study aimed to develop a comprehensive computational model of hiPSC‐CMs to capture the essential cellular electromechanics and finally validate the model against hiPSC‐CMs *in vitro* data. Accordingly, a reparametrized version of a cardiac myofilament model proposed by Rice et al. (2008), with a new passive force formulation, has been integrated into a hiPSC‐CM electrophysiology model by Paci et al. (2020). We explored and validated the capability of our model through simulations of AP and CaT biomarkers, the key electrophysiological currents and intracellular concentrations, the contraction–relaxation velocity, fractional cell shortening, generated active tension, aftercontractions, as well as drug‐induced and non‐cardiomyocyte mechanical effects.

## 2 METHODS

### 2.1 The electrophysiology model

Figure S1 in the Supplementary Materials shows the schematics of the hiPSC‐CM‐CE model, outlining cell compartments, ion channels and pumps, and the CE. The model comprises two cellular compartments, which are cytosol and sarcoplasmic reticulum (SR). In Paci2020, both I_f_ and the pre‐upstroke inward component of Na^+^/Ca^2+^ exchanger (I_NCX_) sustain the spontaneous electrical activity. The underlying electrophysiology is described by the classical Hodgkin & Huxley formalism, giving the membrane potential as follows:

(1) $$C\frac{\mathrm{dV}}{\mathrm{dt}}=‐\left(I_{\mathrm{Na}}+I_{\mathrm{NaL}}+I_{\mathrm{CaL}}+I_{f}+I_{K1}+I_{\mathrm{Kr}}+I_{\mathrm{Ks}}+I_{\mathrm{to}}+I_{\mathrm{NaCa}}+I_{\mathrm{NaK}}+I_{\mathrm{pCa}}+I_{\mathrm{bNa}}+I_{\mathrm{bCa}}‐I_{\mathrm{stim}}\right)$$

where *C* denotes cell capacitance, *I_stim_* is the stimulus current, and *V* represents membrane potential. All simulations have been done assuming the temperature at 37°C and with extracellular concentrations of 151, 5.4, and 1.8 mM for Na, K, and Ca^2+^, respectively.

### 2.2 The contractile model

Figure S2 shows a schematic diagram of the CE (Rice et al., 2008), which takes in an adequate cellular machinery while maintaining an admirable balance between mechanistic detail and model parsimony. In Rice CE, to simulate the experimental protocols of internal sarcomere shortening, a series elastic element is considered to enable stretch in the compliant end. Ultimately, to improve the model stability and circumvent sudden changes in sarcomere length velocity, a mass term has been considered.

The development of myofilament active force results from the fraction of cross‐bridges (XBs) which can bind strongly. To elucidate, this process hinges upon the overlay of the thick filament (myosin) and the thin filament (actin). In the hiPSC‐CM‐CE model, the rest length of the sarcomere, at which no passive force exists, has been set 1.9 µm in accord with experimental reports for hiPSC‐CMs (Pioner et al., 2020). Correspondingly, the Newtonian viscosity of the myofilament was set to 0.3% of F_max_ µm^−1^ s^−1^ in line with experimental data (de Tombe & ter Keurs, 1992). In non‐isosarcometric scenarios, the change in sarcomere length follows:

(2) $$\frac{d}{\mathrm{dt}}SL=\frac{{\mathrm{Integral}}_{\mathrm{Force}}+\left({\mathrm{SL}}_{0}‐SL\right)\times vsc}{m}$$

where *SL* is the sarcomere length, *Frc* denotes the developed active force, *vsc* is the viscosity, and *m* is mass. The integral force has been defined by Equation S4 in the supplementary materials.

Importantly, a distinct feature of Rice2008 CE is how the calcium–troponin binding system and thus the XB machinery is handled. There are two types of calcium binding: the regulatory Ca^2+^ binding, which only affects thin‐filament activation, and the apparent Ca^2+^ binding, which influences the cytosolic CaT, in other words, the Ca binding sensed by the cell. Here, we integrated the CE into Paci2020 electrophysiology by (A) subtracting the total troponin concentration from the total buffered Ca^2+^ concentration and (B) subtracting the Ca^2+^ flux toward the myofilaments from the cytosolic Ca^2+^ (the apparent Ca^2+^ binding). The latter is described by:

(3) $$\frac{d}{\mathrm{dt}}\left[{\mathrm{Trop}}_{\mathrm{Apr}}Ca\right]=\left[Troponin\right]\times \frac{d}{\mathrm{dt}}{\mathrm{Trop}}_{\mathrm{Apr}}\left(s\right)$$

(4) $$\frac{d}{\mathrm{dt}}{\mathrm{Trop}}_{\mathrm{Apr}}\left(s\right)=‐\frac{d}{\mathrm{dt}}{\mathrm{SOVF}}_{\mathrm{thin}}\left(s\right)\times {\mathrm{Trop}}_{L}+\left(1‐{\mathrm{SOVF}}_{\mathrm{thin}}\left(s\right)\right)\times \frac{d}{\mathrm{dt}}{\mathrm{Trop}}_{L}+\frac{d}{\mathrm{dt}}{\mathrm{SOVF}}_{\mathrm{thin}}\left(s\right)\times \left({\mathrm{Frct}}_{\mathrm{SBXB}}\times {\mathrm{Trop}}_{L}+\left(1‐{\mathrm{Frct}}_{\mathrm{SBXB}}\right)\times {\mathrm{Trop}}_{L}\right)+{\mathrm{SOVF}}_{\mathrm{thin}}\left(s\right)\times \left(\frac{d}{\mathrm{dt}}{\mathrm{Frct}}_{\mathrm{SBXB}}\times {\mathrm{Trop}}_{L}+{\mathrm{Frct}}_{\mathrm{SBXB}}\times \frac{d}{\mathrm{dt}}{\mathrm{Trop}}_{H}‐\frac{d}{\mathrm{dt}}{\mathrm{Frct}}_{\mathrm{SBXB}}\times {\mathrm{Trop}}_{L}+\left(1‐{\mathrm{Frct}}_{\mathrm{SBXB}}\right)\times \frac{d}{\mathrm{dt}}{\mathrm{Trop}}_{L}\right)$$

As given in Equation (3), the time rate of apparent Ca^2+^ binding to troponin, ${\mathrm{Trop}}_{\mathrm{Apr}}$ is multiplied by the total buffer concentration of troponin, [Troponin], which is 70 µM in our simulations. Also, the flux from cytosolic Ca^2+^ toward the myofilament is given by Equation (4). Here, ${\mathrm{SOVF}}_{\mathrm{thin}}\left(s\right)$ is the thin filament overlap, which is a function of sarcomere length *s*. ${\mathrm{Trop}}_{L}$ and ${\mathrm{Trop}}_{H}$ denote the Ca^2+^ binding to low and high affinity troponin, respectively. Finally, ${\mathrm{Frct}}_{\mathrm{SBXB}}$ is the fraction of strongly attached XBs. The equations and details of the CE used in the hiPSC‐CM‐CE have been fully given in Rice et al. (2008).

Recently, the role of cardiomyocytes to non‐cardiomyocytes ratio has been proven critical in electromechanics of engineered heart tissues (EHTs) derived from hiPSC‐CMs. Similarly, it plays a vital role in the structure/function and therapeutic potentials of hiPSC‐CMs (Iseoka et al., 2018). Notably, the quantity of non‐cardiomyocytes is crucial in generating functional iPSC‐derived EHTs as grafts in cardiac‐regeneration therapy. The EHTs containing 50–70% of cardiomyocytes exhibited stable structures and increased therapeutic potential (Iseoka et al., 2018). To include the effect of the non‐cardiomyocyte components in the model and according to the biphasic trend in contraction–relaxation velocities observed for different cardiomyocyte ratios in EHTs (Figure S7), we introduced a piecewise function as the passive force (Equation 5). We used an exponential first guess for the curve fitting regarding the trends observed in Figure S7 and minimized the error manually, so maximums of simulated contraction–relaxation velocities best replicate the data in Figure S7. Here, the passive force represents the effect of the non‐cardiomyocyte components on the sarcomere dynamics and cell contractions which is defined as follows:

(5) $$F_{\mathrm{passive}}\left(x\right)=\left\{\begin{array}{c} F_{\mathrm{titin}}\left(x\right),\;\;ctn=100\\ \frac{1}{0.54\;\times \;(4.66\;\times \;e^{‐3.05c^{4.9}})}\times \left(F_{\mathrm{titin}}\left(x\right)+F_{\mathrm{collagen}}\left(x\right)\right),\;\;100>ctn\geq 70\\ \left(6\times e^{‐6.17c^{2.91}}\right)\times \left(F_{\mathrm{titin}}\left(x\right)+F_{\mathrm{collagen}}\left(x\right)\right),\;\;70>ctn>0 \end{array}\right.$$

where *c* is equal to *ctn*/100 and ctn indicates the percent of CMs in the EHT, *x* denotes the sarcomere length, and CM is cardiomyocyte.

### 2.3 Reparameterization of the contractile element

The foundation of the development of the Rice et al. CE was *in vitro* rabbit data. Therefore, by adapting XB cycling and calcium‐based thin filament activation parameters, the CE was adjusted to match the literature‐based hiPSC‐CM Ca^2+^ and tension data. The main strategy in reparametrizing the CE was finding a set of parameters with which the model can simulate key contraction‐related hiPSC‐CMs protocols in the reported experimental ranges in 37°C and extracellular Ca^2+^ concentration of 1.8 mM (see Section 2.4). Namely, % of cell shortening at 1 Hz pacing and contraction RT_50_ (the time from contraction peak to half of the relaxation) from Pioner et al. (2020), and the amplitude of developed active tension from Ruan et al. (2016).

The tuned parameters of the CE mainly govern the Ca^2+^ binding to troponin, except for *m* (mass), in Equation (2), which is essentially a tuning parameter related to improving the model response times, and *xbmodsp*, which is the species‐dependent parameter influencing the thin filament regulation and XB cycling and inversely correlates with the size of the organism (Rice et al., 2008). Table 1 gives the modified and original values of the parameters in the CE.

**TABLE 1:** Baseline (Rice et al., 2008) and the modified values of the parameters changed in the CE

Parameter	Baseline value	The hiPSC‐CM‐CE	% of change
K_on_ (s^−1^ mM^−1^)	50 × 10^3^	62.5 × 10^3^	25
K_offL_ (s^−1^)	250	200	−20
K_offH_ (s^−1^)	25	25	0
Perm_50_	0.5	0.6	20
n_perm_	15	11.28	−24.8
K_n‐p_ (s^−1^)	500	550	10
K_p‐n_ (s^−1^)	50	50	0
K_offmod_	1	0.5	−50
m (s^2^ µm^−1^)	5 × 10^−5^	2 × 10^−5^	−60
kxb	120	12	—
xbmodsp	0.2, 1, 1.33	0.2	—

**FIGURE 1:**
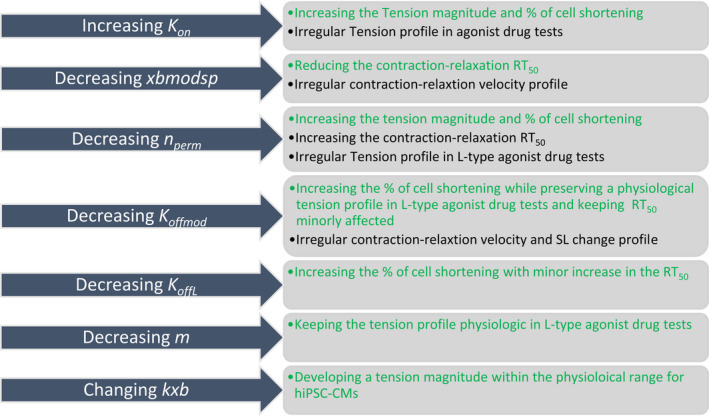
Tuning parameters of the CE and their effect on the hiPSC‐CM‐CE. The green and black texts highlight the positive and negative effects on the mechanical outputs selected for validation, respectively

In this work, we tuned the CE parameters manually, following the adjustments reported in (Zile & Trayanova, 2018), in order to simulate the available experimental data. According to the equations handling the troponin and XB regulations, we first investigated the effect of the changes in the parameters listed in Figure 1 on the mechanical outputs we aimed to simulate. The green and black texts denote the positive and negative effects on the mechanical outputs selected for validation, respectively. We highlight that the effects presented in Figure 1 do not come from a sensitivity analysis, but from the manual tuning of the parameters performed in a sequential manner. We considered ±25% of the CE baseline values as the constraints for the parameter tuning, except for *K_offmod_*, *m*, and *kxb*. *K_offmod_* is a species‐dependent parameter (inversely correlating with the organism's size) and contributes to the total rate for unbinding rate in the high‐ and low‐affinity conditions in regulatory Ca^2+^ binding. The mass, denoted by *m* in Equation (2), improves the stability of the integration of the model equations and avoids rapid changes in muscle shortening velocity in fast muscle release protocols. It is also a parameter to tune and improve the model response times (Rice et al., 2008). Markedly, similar limitations and assumptions considered in this work have been reported previously for Rice CE reparameterizations (Campbell et al., 2008; Zile & Trayanova, 2017).

Furthermore, we changed the value of *kxb*, the tension scaling parameter in the reparameterization. In the hiPSC‐CM‐CE model, tension is scaled to obtain the final simulated tension from the normalized active tension as follows:

(6) $$Tension=kxb\times F_{\mathrm{active}}$$

where $F_{\mathrm{active}}$ is calculated by Equation 35 in Rice et al. (2008), and *kxb* is the scaling constant that corresponds to the total number of XBs and the fraction of cycling XBs in the force‐generating state at any time, as also reported in Land et al. (2012).

In the literature, this scaling constant has been treated either as an original experimental value or a model‐based parameter whose value depends upon other experimental values. As an example of the latter, in the Niederer‐Hunter‐Smith (NHS) model (Niederer et al., 2006), the equivalent parameter is 56.2 kPa. However, the value is set to 100 kPa in later whole‐organ models (Niederer & Smith, 2009). In Rice original CE model, *kxb* ≈ 120 kPa. Also, Land et al. set this scaling constant at 120 kPa consistently with previous models and with the higher end values for maximum tension and twitches (Land et al., 2012).

The values reported experimentally for the *kxb* in the literature range within multiple orders of magnitudes. Blanchard et al. reported maximum tension in mouse muscle strips to be 12.96 kPa (Blanchard et al., 1999). Stuyvers et al. indicated the maximal stress is around 17 kPa for mouse muscle with 1.9 µm of sarcomere length (Stuyvers et al., 2002). Palmer et al. also reported comparable maximum activated tension for mice isometric analysis around 14 kPa (Palmer et al., 2004). However, some twitch transient data peak at 50 kPa (Stull et al. 2002) and other results go up to 112 kPa for maximum developed tension (Kreutziger et al., 2011).

Peak active tensions experimentally reported for hiPSC‐CMs range from ~1.2 to ~45 kPa (Pioner et al., 2020; Yang et al., 2018). Specifically, Pioner et al. have reported 18.6 ± 2.5 as the maximal developed tension for in hiPSC‐CM myofilaments (Pioner et al., 2020). However, there are other studies that report an order of magnitude lower peak twitches for hiPSC‐CM‐CEs (Rodriguez et al., 2014; Ruan et al., 2016). In line with these findings, we set *kxb* = 12 kPa, placing the peak tension within the experimental data range for hiPSC‐CMs and thus resulting in the final simulated 0.055 kPa active tension.

Lastly, we performed the sensitivity analysis (Figure S3) to investigate the hiPSC‐CM‐CE behavior regarding the mechanical biomarkers following the method detailed in Romero et al. (2011). For each biomarker, b, and model parameter, p, the percentage of change D_c,p,a_, sensitivity S_c,p_, and relative sensitivity r_c,p_, are defined as follows:

(7) $$D_{b,p,m}=\frac{\left(b_{p,m}‐b_{\mathrm{ctl}}\right)}{b_{\mathrm{ctl}}}\times 100$$

(8) $$S_{b,p}=\frac{{\Delta D}_{b,p,m}}{\Delta m}=\frac{D_{b,p,+15\%}‐D_{b,p,‐15\%}}{0.3}$$

(9) $$r_{b,p}=\frac{S_{b,p}}{{\left|S_{b,p}\right|}_{max,b}}$$

### 2.4 In vitro data for the contractile element reparameterization

The experimental ranges obtained from hiPSC‐CMs based on which the CE of the model has been reparametrized were the percent of cell shortening (preserved cell contractility) (Pioner et al., 2020), the contraction–relaxation RT_50_ (Pioner et al., 2020), and the tension magnitude (Ruan et al., 2016).

In Pioner et al. (2020), the supplementary materials detail the process of obtaining the differentiated cardiomyocytes and their long‐term maturation as single cells on nanotopographic (nanopattern) substrata. An inverted microscope integrated into a video‐based edge detection system has been used for visualization of single hiPSC‐CMs fractional shortening (% of cell shortening). The cells were paced at 0.5, 1, or 2 Hz using field stimulation and were perfused at 37°C, with Tyrode solution.

In Ruan et al. (2016), undifferentiated hiPSCs were obtained from a lung fibroblast cell line. After trypsinization of the hiPSC‐derived cardiomyocytes into single cells, engineered heart tissue structures were made of a collagen‐based three‐dimensional scaffold encapsulating the single cells. The 2‐mm‐long sections constructs were attached to a force transducer and a length controller. Using LabView software, length and force signals of spontaneously contracting constructs were digitally recorded. The authors assumed a circular cross‐section for the preparations and normalized the force to the cross‐sectional area of the constructs. The diameter was measured at non‐strained lengths, and the area was calculated accordingly. The *in vitro* data were obtained when the solution temperature was maintained at 37°C.

In Iseoka et al. (2018), using magnetic‐activated cell sorting, the scaffold‐free hiPSC‐CM EHTs at different ratios of cardiomyocytes (25%, 50%, 70%, or 90%) were generated. Using a camera‐based motion analysis setup, the contractile features of the EHTs were investigated. Table 2 gives a concise map of the model reparameterization with attention to the hiPSC‐CMs experimental results reported in the cited papers.

**TABLE 2:** hiPSC‐CM experimental papers used for the reparameterization of the model

#	Experimental paper	[Ca^2+^]_e_	Temperature (°C)	Biomarkers used for the reparameterization
1	(Pioner et al., 2020)	1.8 mM	37	% of cell shortening,
2	(Clark et al., 2021)	1.8 mM	37	Contraction RT_50_
3	(Yang et al., 2018)	1.8 mM	37	% of cell shortening, contraction RT_25_,
4	(Ruan et al., 2016)	1.8 mM	Mechanical measurements and drug tests at 37, Histological measurements and microscopy at room temp.	Active tension amplitude and inotropic effects of Verapamil and Bay‐K 8644
5	(Iseoka et al., 2018)	N/A	37	Inotropic effects of non‐cardiomyocytes on the mechanical outputs of hiPSC‐CMs
6	(Hayakawa et al., 2014)	N/A	37	Contraction velocity profile
7	(Rodriguez et al., 2014)	N/A	37	Contraction velocity profile

Note

[Ca^2+^]_e_ denotes the extracellular Ca^2+^ concentration.

### 2.5 Drug tests protocols

The occurrence of aftercontractions (Nguyen et al., 2017; Novak et al., 2012) is caused by abnormalities in the electrophysiology, e.g. delayed (DADs) and early afterdepolarizations (EADs). To trigger EADs, we blocked *I_Kr_* by 95%. Since the Paci2020 model responds to a strong *I_Kr_* block with a remarkable APD prolongation but does not develop EADs (Paci, Koivumäki, et al., 2021), to evaluate the occurrence of aftercontractions, we tested *I_Kr_* block on few illustrative model variants capable of generating EADs for strong *I_Kr_* block. These models were extracted from an *in silico* population previously generated using the Paci2020 model as a baseline and modulating maximum conductances/currents of *I_Na_*, *I_NaL_*, *I_CaL_*, *I_f_*, *I_to_*, *I_Kr_*, *I_Ks_*, *I_K1_*, *I_NaCa_*, *I_NaK_* and *I_pCa_* in the range [0.5, 2], as done in Paci, Pölönen, et al. (2018).

Furthermore, the drug‐induced inotropic effects were investigated by simulating the effect of 90 nM of Verapamil (as the *I_CaL_* inhibitor) and 1 µM of Bay‐K8644 (the *I_CaL_* agonist). We simulated Verapamil administration with a simple pore‐block model, setting the IC_50_ and Hill coefficients of the involved blockers as given in Table S1 in the supplementary materials. Similarly, the effect of 1µM of Bay‐K8644 on the model was studied, setting 17.3 nM and 1.25 as the EC_50_ and Hill coefficient of *I_CaL_* blocker, respectively (Bechem & Hoffmann, 1993; Rae & Calixto, 1989).

## 3 RESULTS

### 3.1 Reparameterization: contractility

**FIGURE 2:**
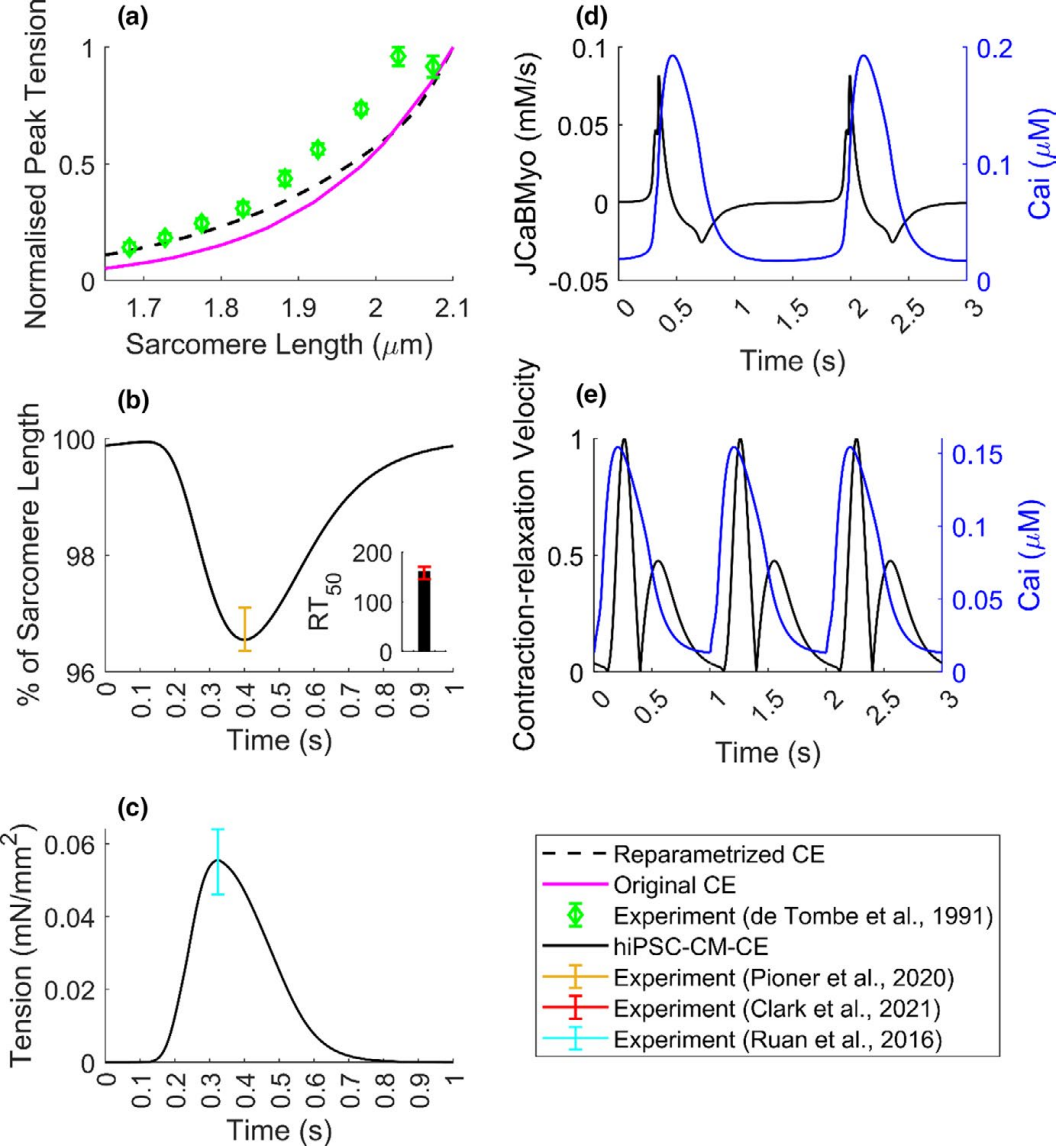
Mechanical biomarkers simulated by the hiPSC‐CM‐CE model. (a) Normalized peak tensions vs. SL. (b) % of cell shortening and contraction RT_50_ (time from peak contraction to 50% relaxation) at 1 Hz pacing. (c) The simulated tension profile at spontaneous beating. (d) The binding flux of Ca^2+^ toward the myofilament (JCaBMyo) and the CaT. (e) Normalized motion waveform (contraction–relaxation velocity) and the CaT at 1 Hz pacing

The main results of integrating original Rice CE into the Paci2020 electrophysiology are shown in Figure S4. The uncalibrated version failed to simulate the main mechanical results within the *in vitro* ranges. We also studied the role of mechanical feedback (Figure S5). Results indicate that the strong coupling has a significant effect on the cytosolic Ca^2+^ concentration. Compared with the weakly coupled condition, the CaT peak was decreased by 20.3%, and the simulated active tension consequently by 52.2%. This behavior is consistent with the results of the mechanical feedback study done by a hV‐CM ionic model integrated into Rice CE (Zile & Trayanova, 2016). The strong coupling term comes against weak coupling, where there is no mechanical feedback. In other words, by strong coupling, we mean incorporating myofilament feedback on calcium dynamics which was done using a dynamic term for troponin buffering of intracellular calcium (Equation 4) using the approach in Rice et al. (2008). Of note, the profile and magnitude of JCaBMyo (Figure 2d) are consistent with the corresponding simulations by other mathematical CE models (Negroni & Lascano, 2008; Negroni et al., 2015).

For the skinned version of the CE, the steady‐state tension vs. sarcomere length (SL) relationship is one of the experimental characterizations that significantly influences the model development. As can be seen in Figure 2a, in comparison with the original CE, there is an improvement in the physiologic behavior in the reparametrized version in terms of fitting to the reference experimental data obtained from cat trabeculae (De Tombe & Ter Keurs, 1991).

In line with cell shortening data obtained for hiPSC‐CMs in Pioner et al. (2020), our model simulates the cell shortening within experimental range at 1 Hz pacing (Figure 2b). Also, at 1 Hz pacing, RT_50_ was simulated 161 ms, which is in the experimental range reported for hiPSC‐CMs (145.9–170.1 ms (Clark et al., 2021)). Figure 2c shows the tension amplitude simulated by the hiPSC‐CM‐CE, in spontaneous condition, which locates within the experimental range reported for hiPSC‐CMs at matching conditions of 1.8 mM extracellular Ca^2+^ and 37°C (Figure 2c) (Ruan et al., 2016). Figure 2d shows the flux of Ca^2+^ toward the myofilament and the corresponding CaT trace. Furthermore, Figure 2e illustrates the normalized contraction velocity curve, which recapitulates the standard shape and the experimental records for hiPSC‐CMs obtained via traction force microscopy in Hayakawa et al. (2014).

Figure S3 in the supplementary material shows a sensitivity analysis map of the hiPSC‐CM‐CE model regarding the target mechanical outputs. The relative sensitivity of the biomarkers (fractional cell shortening, active tension peak, and RT50) is shown on a 0–1 scale as a response to 15% of change of the parameters on the y‐axis. For example, *n_perm_* has the most intense inverse effect on ATpeak and RT50, meaning, by decreasing *n_perm_* the biomarker values increase. The results indicate that each simulated contractile biomarker, specifically % of cells shortening (Figure S4d), is heavily influenced by *n_perm_*; the parameter that represents the non‐linear function of nearest‐neighbor cooperativity in myofilament Ca^2+^‐based activation in cycling XBs in Rice CE.

### 3.2 Electrophysiological properties of the hiPSC‐CM‐CE model

In Table 3, we reported the biomarkers simulated by the Paci2020 + the original Rice CE (i.e. before the CE calibration) model and the hiPSC‐CM‐CE model, together with the experimental biomarkers previously summarized in Paci, Pölönen, et al. (2018). The simulations were done in the spontaneous beating condition until the steady‐state.

**TABLE 3:** Biomarkers computed on simulated spontaneous APs and CaTs and their comparison with Paci2020 model, Paci2020 + Original rice CE model (i.e. before the calibration of the contractile element) and the experimental values (Paci et al., 2020; Paci, Pölönen, et al., 2018)

No.	Biomarker	Paci2020	Paci2020 + Original Rice CE	hiPSC‐CM‐CE	Exp. value (Mean ± SD)
1	APA (mV)	102	105	103	104 ± 6
2	MDP (mV)	−74.9	−75.3	−75.0	−75.6 ± 6.6
3	AP CL (ms)	1712	1559	1644	1700 ± 548
4	dV/dt max (V/s)	20.5	14.0	23.9	27.8 ± 26.3
5	APD_10_ (ms)	87.0	*109.5*	95.0	74.1 ± 26.3
6	APD_30_ (ms)	224	*259*	238	180 ± 59
7	APD_90_ (ms)	390	421	403	415 ± 119
8	AP Tri	2.8	3.2	2.9	2.5 ± 1.1
9	CaT DURATION (ms)	691	681	693	805 ± 188
10	CaT tRise_10, peak_ (ms)	184	*136*	163	270 ± 108
11	Cat tRise_10,50_ (ms)	54.9	39.2	46.2	82.9 ± 50.5
12	CaT tRise_10,90_ (ms)	118	*86*	102	167 ± 70
13	CaT tDecay_90,10_ (ms)	341	349	343	410 ± 100

Note

AP biomarkers listed are: APA (AP amplitude), MDP (maximum diastolic potential), CL (cycle length), dV/dt max (maximum upstroke velocity), APD_10_ and APD_30_ and APD_90_ (AP duration at 10, 30, 90% of repolarization, respectively), AP Tri (AP triangulation index). And CaT biomarkers are DURATION (duration of the transient), tRise_10, peak_ (time to peak), tRise_10, 50_ and tRise_10, 90_ (rise time from 10 to 50% and 90% of maximum threshold, respectively), and tDecay_90,10_ (decay time from 90 to 10%). The out‐of‐range values are in italics. The third column is taken directly from the original Paci2020 publication (Paci et al., 2020).

**FIGURE 3:**
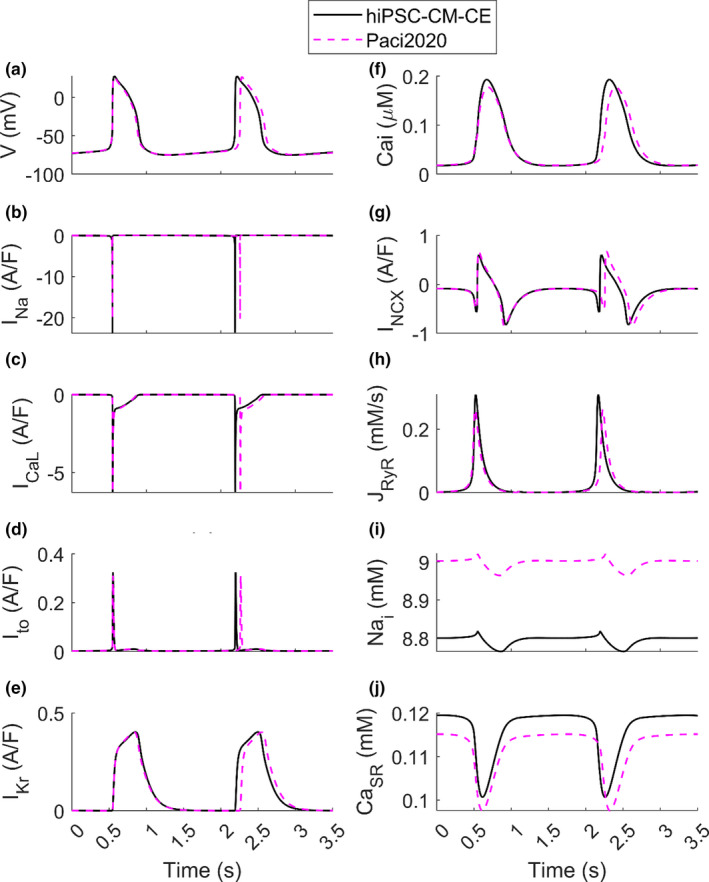
Simulated action potentials and ionic currents of the hiPSC‐CM‐CE model vs. Paci2020 (Paci et al., 2020) in spontaneous beating in the steady‐state condition. (a) Membrane potential. (b) Fast Na^+^ current (I_Na_). (c) L‐type Ca^2+^ current (I_CaL_). (d) Transient outward K^+^ current (I_to_). (e) Rapid delayed rectifier K^+^ current (I_Kr_). (f) Cytosolic Ca^2+^ concentration (Ca_i_). (g) Na^+^/Ca^2+^ exchanger (I_NCX_). (h) Ca^2+^ release from sarcoplasmic reticulum (J_RyR_). (i) Cytosolic Na^+^ concentration (Na_i_). (j) Sarcoplasmic Ca^2+^ concentration (Ca_SR_)

Table 3 presents that the integration of the original CE into the ionic model moved 4/13 biomarkers out of the range and the reparameterization of the CE restored them (column Paci2020 + Original Rice CE vs. hiPSC‐CM‐CE). Since the hiPSC‐CM‐CE model inherits its electrophysiology from the Paci2020 model, which does not simulate the contractile function of hiPSC‐CMs, we compared the simulated AP, CaT, and ionic current traces of the two models (Figure 3).

### 3.3 Validation: fractional shortening, drug‐induced, and arrhythmogenic effects

To validate the hiPSC‐CM‐CE model, we compared the simulated contraction with *in vitro* data from Yang et al. (2018) that was not used in the model parameterization process. Furthermore, the effects of 90 nM of Verapamil (antiarrhythmic drug class IV) and 1µM of Bay‐K 8644 (L‐type Ca^2+^ channel agonist) on the model outputs were simulated.

**FIGURE 4:**
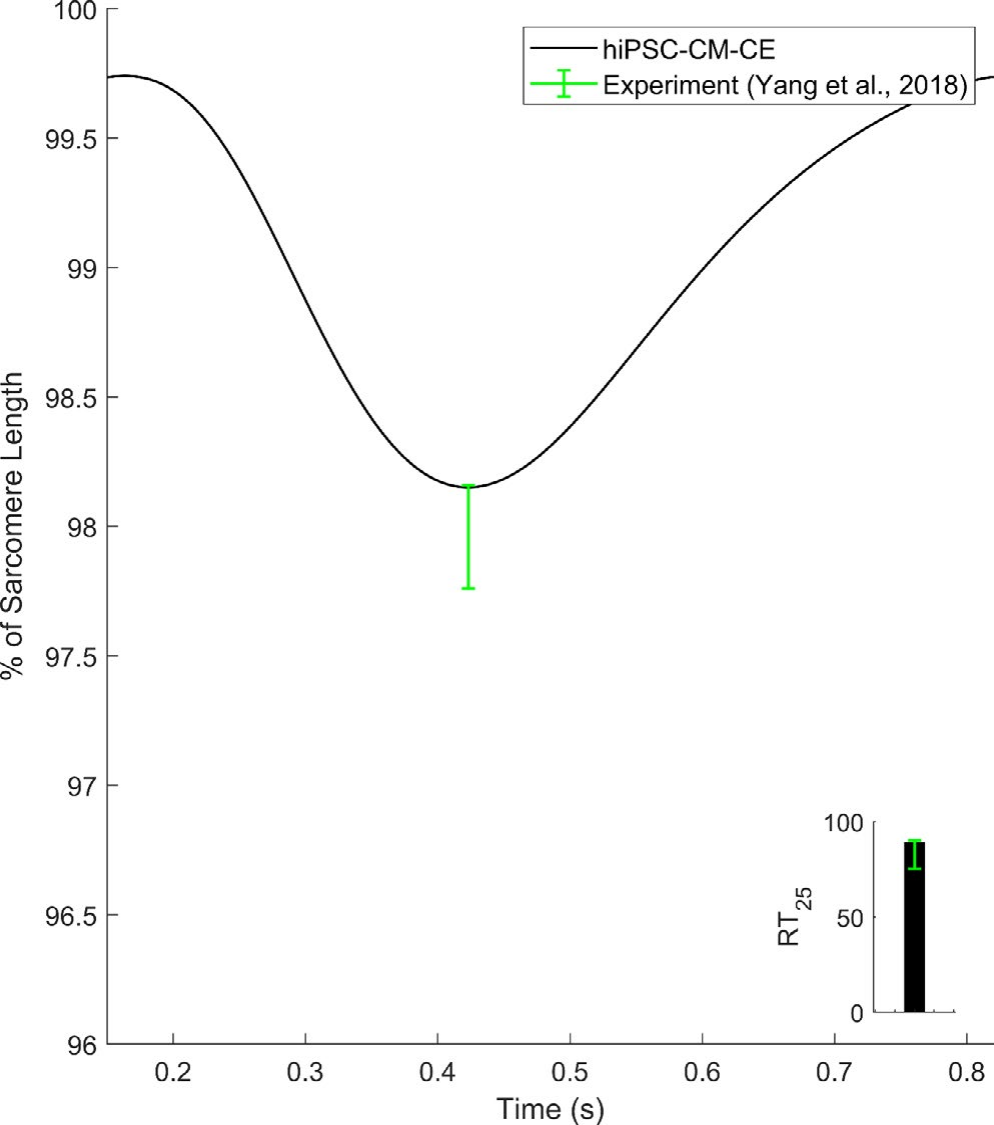
Percent of cell shortening and the contraction RT_25_ (time from peak contraction to 50% of relaxation) simulated by the hiPSC‐CM‐CE model

As Figure 4 shows, the hiPSC‐CM‐CE mechanical results at 1.5 Hz pacing place within the ranges of experimental contraction biomarkers reported for hiPSC‐CM single cell lines at 1.8 mM extracellular Ca^2+^ and 37°C (Yang et al., 2018). The simulated fractional cell shortening and the contraction RT_25_ are 1.86% and 89 ms, located within the reported *in vitro* intervals of 2.04 ± 0.2% and 75.2–90.3 ms, respectively.

The effect of 90 nM Verapamil was simulated as a multichannel action reference compound, which was selected according to human‐based data (Kramer et al., 2013). On the other hand, the effect of 1 µM of Bay‐K 8644, for which we expect a positive inotropic effect as in Ruan et al. (2016), has been simulated as an agonist drug compound that influences only *I_CaL_*.

**FIGURE 5:**
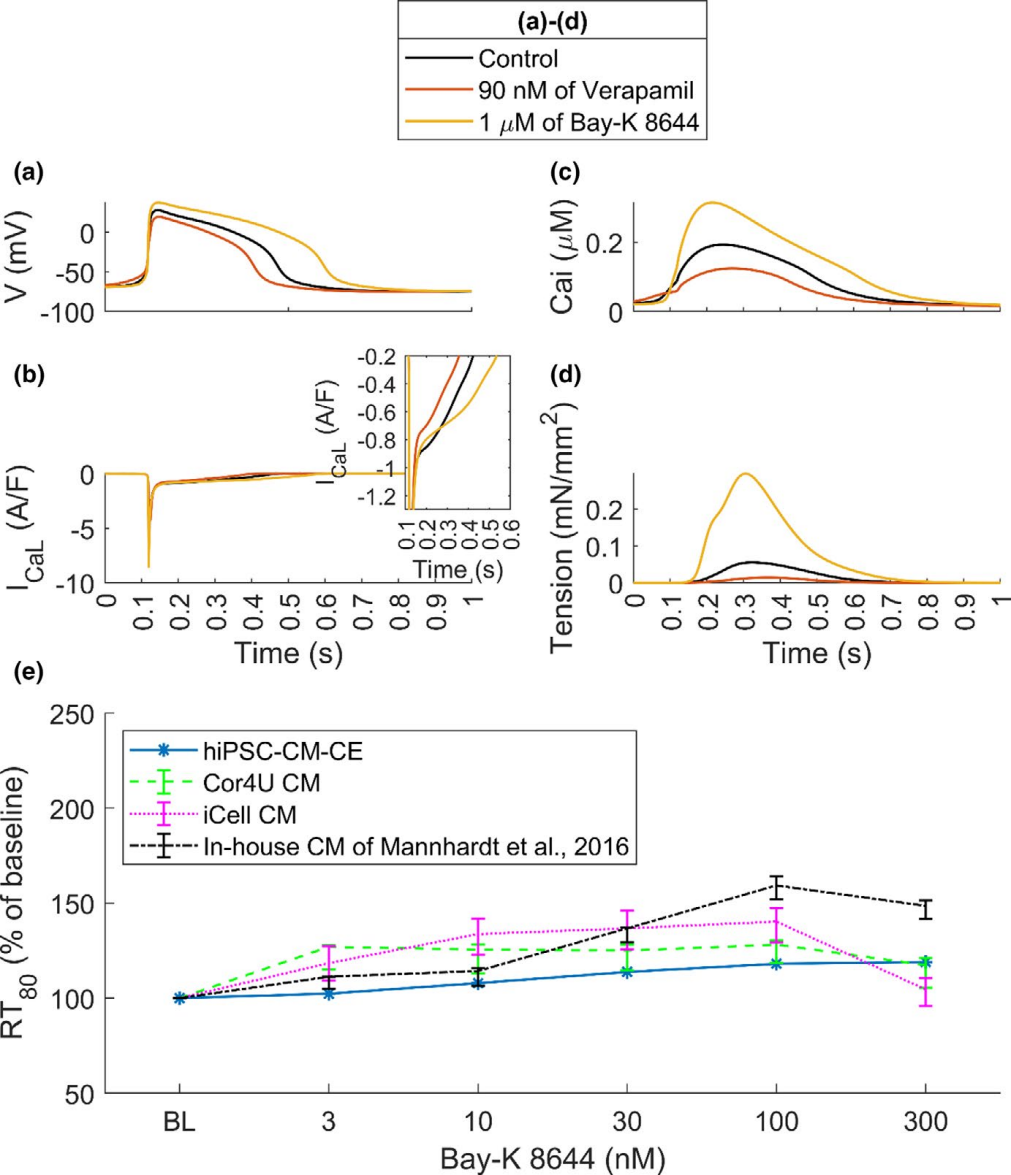
Electrophysiology and contractility of the hiPSC‐CM‐CE in control and drug modes. (a) Action potentials. (b) L‐type Ca^2+^ currents. (c) Cytosolic Ca^2+^ transients. (d) Active tensions. (e) RT_80_ (time from peak contraction to 80% of relaxation) results of the model in response to different concentrations of Bay‐K 8644 and the in vitro data obtained from different hiPSC‐CMs. Cor4U and iCell are commercial cardiomyocytes the data of which have been acquired from Mannhardt et al. (2016). Cases (a–d) show data at spontaneous condition, and case (e) shows the model results at 1.5 Hz pacing. BL: Baseline. Of note, the same tests presented in panels (a–d) were also performed in paced conditions (1 Hz), without observing noteworthy differences with the results presented in this figure

The hiPSC‐CM‐CE simulation results show that Bay‐K 8644 prolongs the AP (Figure 5a) and brings an increase in cytosolic Ca^2+^ (Figure 5c), and thus an elevated active tension (Figure 5d). These findings are in line with the AP prolongation (Sicouri et al., 2007) and positive inotropic effect reported *in vitro* for Bay‐K 8644 (Ruan et al., 2016).

As is observable in Figure 5d, in accord with reports classifying Verapamil as a negative inotropic drug in hiPSC‐CMs (Ruan et al., 2016) and hV‐CMs (Nguyen et al., 2017), our results correctly replicate Verapamil‐induced effects on the mechanical outputs. Finally, the relaxation kinetics of the hiPSC‐CM‐CE model, analyzed using RT_80_ (time from peak contraction to 80% of relaxation), follows the experimental trends and ranges reported for commercial and lab‐based hiPSC‐CMs as illustrated in Figure 5e.

**FIGURE 6:**
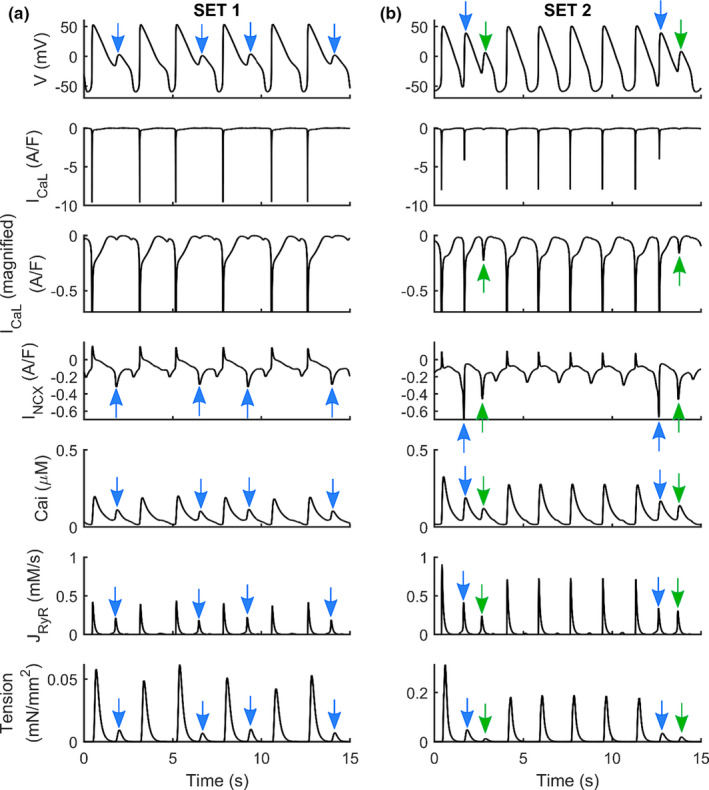
Action potentials, L‐type Ca^2+^ currents (I_CaL_), Na^+^/Ca^2+^ exchangers (I_NCX_), Calcium Transients, Ca^2+^ releases from the sarcoplasmic reticulum (J_RyR_), and active tensions simulated for two sets of parameters (SET1 and SET2) used to generate models which develop EADs (a) and (b), using hiPSC‐CM‐CE as baseline. Blue arrows show almost full anticipated APs due to the strong first inward I_NCX_ activation. Then, a second I_NCX_ activation triggers the second EADs where we observe an I_CaL_ reactivation up to −0.22 pA/pF (green arrows). The scales of tensions are different. In the third line, we show a magnification of the I_CaL_ traces, highlighting I_CaL_ reactivation

Concurring with experimental results, the induction of abnormalities in electrical repolarization (EADs) can occur due to the use of drugs known to block the *I_Kr_* current, namely, the class III antiarrhythmic Dofetilide (Guo et al., 2011) and the abnormalities in diastolic depolarization (DADs) can occur due to Isoproterenol (Novak et al., 2012). Those arrhythmogenic phenomena can also lead to contractile irregularities detectable in the form of aftercontractions, as has been shown previously in hV‐CMs (Nguyen et al., 2017) and hiPSC‐CMs (Novak et al., 2012). Figure 6 shows selected electrophysiologic and mechanical traces simulated by the hiPSC‐CM‐CE after we tuned its maximum conductances and currents with two different coefficient sets, namely SET1 and SET2, that had triggered EADs in the Paci2020 model, as response to 95% *I_Kr_* block. Of note, the original Paci2020 model (as its predecessor Paci2018) responds to a strong *I_Kr_* block with an extreme APD prolongation but no EADs (Paci, Koivumäki, et al., 2021; Paci, Pölönen, et al., 2018). However, experimentally calibrated populations of models generated by using Paci2020 or Paci2018 models contain models that can produce EAD as a response to *I_Kr_* block or more in general during *in silico* tests of multichannel drugs with a substantial effect on hERG (Paci et al., 2018, 2020). As shown in Figure 6, 95% *I_Kr_* block triggers EADs in SET1 and SET2 and the consequent aftercontractions. In Figure 6, both in SET1 and SET2, we observe APD prolongation due to the *I_Kr_* block and the subsequent alterations in CaTs due to anomalous Ca^2+^ releases from SR (see the arrows in *J_RyR_*). On the one hand, these releases increase the cytosolic Ca^2+^ concentration, thus triggering aftercontractions. On the other hand, they activate the inward *I_NCX_* that depolarizes the membrane potential during its repolarization, which triggers EADs (Paci et al., 2021; Priori & Corr, 1990; Szabo et al., 1994). In SET2, we even observe consecutive EADs and aftercontractions. In this case, the first inward *I_NCX_* activation is so strong that triggers almost a full anticipated AP (blue arrows). Then, a second *I_NCX_* activation triggers the second EADs, where we observe an *I_CaL_* reactivation up to −0.22 A/F (green arrows). Furthermore, we replicated the Verapamil and Bay‐K 8644 tests also on SET1 and SET2 (Figure S6), observing in both cases a negative inotropic effect for Verapamil and a positive inotropic effect for Bay‐K 8644 consistent with the results in Figure 5.

### 3.4 Exploring the impact of non‐cardiomyocytes

**FIGURE 7:**
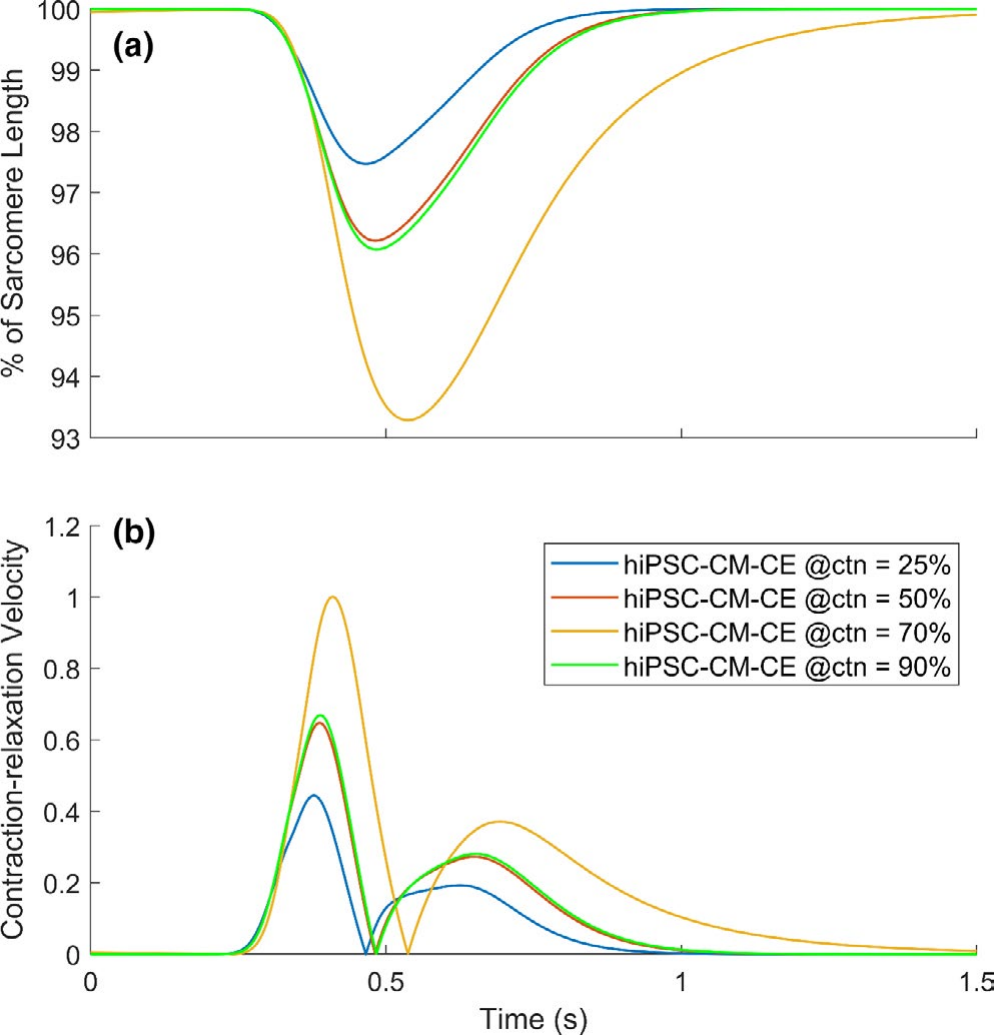
Simulated percent of cell shortenings (a) and (b) contraction–relaxation velocities at different percents of cardiomyocytes in the engineered heart tissue (ctns), normalized over the maximum value simulated for ctn = 70%

The pivotal role of non‐cardiomyocytes in the electromechanics of hiPSC‐CMs, expressly the mechanical outputs, has been confirmed previously (Iseoka et al., 2018). Here, following the biphasic trend in contraction–relaxation velocities observed for different ctn ratios in EHTs (Figure S7), we defined the CE passive force as a piecewise function (Equation 5). The implementation of a new passive force to the CE led to a correct categorization of the inotropic effects of non‐cardiomyocytes (Figure 7). This is considered as an inter‐scale analysis of the behavior of the hiPSC‐CM tissues provided by the hiPSC‐CM‐CE simulations. In detail, inter‐scale means that simulations were done based on a single cell (0D) framework simulating and predicting in a simple way the behavior of a multicellular (1D or 2D) domain.

When changing the ctn, the model simulated positive and negative inotropic effects according to the *in vitro* data reported in Iseoka et al. (2018) for contraction–relaxation velocity and cell deformation. The highest deformation distance, which also can represent the contractile force, was simulated for EHTs comprising 70% of cardiomyocytes (Figure 7a). Moreover, our results show that the contraction–relaxation velocities increase by increasing the cardiomyocyte portion (Figure 7b). Velocities simulated at 90% of ctn were smaller than those simulated for EHTs comprising 70% cardiomyocytes, as reported experimentally in Iseoka et al. (2018). Importantly, the model predicts the lowest contractile performance in accord with the reported non‐cardiomyocyte effects indicating the highest inhibition of electrical propagation for ctn = 25% (Iseoka et al., 2018).

## 4 DISCUSSION

This work proposes an electromechanical hiPSC‐CM model obtained by coupling the Paci2020 ionic model, and the reparametrized Rice2008 CE model, while also adding a new passive force handling. The mechanical behavior of the model has been independently validated by key contraction protocols reported experimentally for hiPSC‐CMs. Notably, the inotropic effect of different multichannel action reference drugs and arrhythmogenic effects, in terms of aftercontractions, have been accurately predicted by the hiPSC‐CM‐CE model. The simulations also correctly predicted the dynamics of the contribution of non‐cardiomyocytes to hiPSC‐CM tissues. Overall, this study provides a reliable mechanistic framework for future studies on hiPSC‐CMs and the functions of immature cardiac cells.

### 4.1 hiPSC‐CM‐CE electromechanics

The capability to simulate key in vitro AP and CaT biomarkers is necessary for *in silico* cardiomyocyte models. The hiPSC‐CM‐CE model recapitulates all the AP and CaT biomarkers within the experimental ranges reported in (Paci et al., 2020; Paci, Pölönen, et al., 2018). Furthermore, Table 3 signifies the impact of CE integration and reparameterization regarding the electrophysiological biomarkers. Notably, the reparameterization of the CE could restore the four electrophysiological biomarkers that were out‐of‐range when the original Rice CE was used instead of the reparametrized one.

The biochemical XB‐related phenomena can be represented more accurately by the mathematical myofilament models consisting of many states, yet the computational cost is significant as well. Thus, maintaining a balance between biophysical accuracy and computational efficiency is the primary goal of current myofilament dynamic models. Given this, we selected Rice et al. (2008) model for this research as the essential calcium‐based activation and sarcomere length‐based sensitivity mechanisms are incorporated by this contractile machinery. A thorough review on this subject has been given earlier (Trayanova & Rice, 2011). Correspondingly, our main motivation to reparametrize the original Rice CE was enabling the model to accurately recapitulate a broad range of electrophysiological and mechanical readouts as the Paci2020 + original Rice CE could not (Figure S4 and Table 3).

The assessment of the contractility is crucial when developing a mathematical cell model of cardiac electromechanics (Campbell et al., 2008; Forouzandehmehr et al., 2020; Margara et al., 2020, 2021; Negroni et al., 2015; Pioner et al., 2020; Rice et al., 2008; Tran et al., 2017; Zile & Trayanova, 2017). In that context, the tension–SL relationship is not only a standard experimental protocol in myofilament studies (Negroni et al., 2015; Rice et al., 2008) but also serves as a measure for evaluating the CE mathematical models. The tension–SL relationship in Figure 2a shows that the reparametrized CE was able to replicate an improved tension–SL curve regarding the experimental ranges obtained for cat trabeculae.

Correspondingly, another key mechanical index here is fractional cell shortening (Figure 2b), which often is considered as a representative of the contractile capacity (Pioner et al., 2020). At 1 Hz pacing, the hiPSC‐CM‐CE has successfully developed % of cell shortening within the experimental range reported for hiPSC‐CMs at 1.8 mM of extracellular Ca^2+^ and 37. Also, the simulated times from peak contraction to 50 and 75% of relaxation (RT_50_ and RT_25_, respectively), as measures for relaxation kinetics, show that the model sits within a desirable domain in terms of mechanical outputs and sarcomere dynamics. It is also worth mentioning that the hiPSC‐CM‐CE model simulates the tension magnitude within the hiPSC‐CMs experimental range at 1.8 mM of extracellular Ca^2+^ and 37 (Figure 2c) (Ruan et al., 2016). These properties endorse the model as a capable mathematical base for future works on modeling contractility and the relevant dysfunctions.

Finally, another essential contractility index routinely reported in experimental myofilament studies (Hayakawa et al., 2014; Rodriguez et al., 2014) is referred to as motion waveform or contraction–relaxation velocity. Lately, the role of decreased relaxation velocity and prolonged relaxation duration in the characterization of diastolic dysfunction has been actively investigated (Hayakawa et al., 2014). Also, the study of cardiac pathologies such as ischaemic diseases, hypertension, and rare genetic disorders are associated with the contraction–relaxation velocity and duration (Hayakawa et al., 2014). The hiPSC‐CM‐CE replicates physiological contraction–relaxation velocity profiles (Figure 2e) consistent with the experimental findings for hiPSC‐CMs (Hayakawa et al., 2014; Rodriguez et al., 2014). Therefore, our model is suitable for simulating the effects of the aforementioned disorders as mentioned above on the contraction–relaxation velocity profiles.

The fundamental role of non‐cardiomyocytes in the electromechanics, function, and therapeutic potential of hiPSC‐CMs tissues has been proved (Iseoka et al., 2018). Notably, in producing functional hiPSC‐derived EHTs as platforms in cardiac‐regeneration therapy, the quantity of non‐cardiomyocytes is vital. The hiPSC‐CM EHTs comprising 50–70% of cardiomyocytes have shown stable structures and augmented the therapeutic potential (Iseoka et al., 2018). As an inter‐scale capability, the hiPSC‐CM‐CE featuring this passive force simulated the contraction–relaxation velocity and cell shortening trends observed experimentally (Figure S7). This also indicates a correct classification of the inotropic effects of non‐cardiomyocytes in hiPSC‐CM tissues simulated on account of the new passive force introduced to the CE. Furthermore, the ctn‐based variations in the simulated positive and negative inotropic effects for motion waveform (Figure 7b) and fractional cell shortening (Figure 7a) are consistent with the corresponding experimental data reported in Iseoka et al. (2018). The model fittingly predicts the lowest contractile potential according to the underlying biophysics behind the non‐cardiomyocyte effects demonstrating the highest inhibition of electrical propagation for ctn = 25% (Iseoka et al., 2018).

### 4.2 hiPSC‐CM‐CE and drug‐induced effects

The pharmaceutical industry and research consider the inotropic and pro‐arrhythmic liabilities as their main concerns (Laverty et al., 2011). Congruently, evaluating the potentials of new drug candidates designed to target cardiac electromechanics is a must in the early phases of drug discovery (Nguyen et al., 2017). Computational approaches have been proved to be a promising opportunity for fast and inexpensive drug screenings and accurate prediction of clinical hazards (Li et al., 2019). Nevertheless, the inotropic risk assessments based on *in silico* approaches are greatly missing. To study the drug‐induced influences on hiPSC‐CMs electromechanics, we have presented a model validated against experimental data reported for relevant drugs. The correct classification of negative and positive inotropic outcomes for the selected reference compounds and the mechanism behind the observed final contractility are captured by hiPSC‐CM‐CE simulations.

The chosen drugs in this study are reference compounds having different pro‐arrhythmic profiles and distinguished clinical results. Verapamil is not classified by CredibleMeds (Woosley et al., 2021), and it is included in the “No TdP risk” category in the *in silico* model‐based ranking by Passini et al. (2017) (Paci et al., 2020). Bay‐K 8644 prolongs action potential duration resulting in prolongation of the QT interval (Sicouri et al., 2007) and is connected with an increase in TdP risk (Sicouri et al., 2010).

The electromechanical coupling correctly replicates the APD prolongation induced by Bay‐K 8644. Consequently, an increase in cytosolic Ca^2+^, and therefore an elevated active tension (Figure 5d) has also been correctly predicted by the model. These results are consistent with positive inotropic results reported for Bay‐K 8644 (Ruan et al., 2016; Sicouri et al., 2007). Markedly, the hiPSC‐CM‐CE accurately predicts the prolonged relaxation due to Bay K‐8644 (negative lusitropic effect). Of note, an essential indicator for proarrhythmic cardiotoxicity is the drug‐induced upsurge in APD_90_, the action potential duration at 90% of its repolarization (Mannhardt et al., 2016). The Bay K‐8644 results simulated here, which follows the traces reported *in vitro* (Figure 5e), confirm that relaxation time RT_80_ of hiPSC‐CMs might be a fit replacement parameter for repolarization time or APD_90_, as also suggested earlier (Mannhardt et al., 2016).

Verapamil is a multichannel action compound blocking a number of currents, namely *I_Kr_*, *I_CaL_*, and *I_NaF_* (Kramer et al., 2013). Markedly, the CaT is mainly affected by the *I_CaL_* block (Figure 5b,c), leading to a negative inotropic effect (Figure 5d) which is consistent with experimental reports of inotropic effects of Verapamil in hiPSC‐CMs (Ruan et al., 2016) and hV‐CMs (Nguyen et al., 2017). Notably, the depressant effect of Verapamil on myocardial contractility pose a potential risk of using it for subjects having severe left ventricular dysfunction and, therefore, it is generally prohibited in such cases (Margara et al., 2021). The simulated Verapamil negative inotropic effect (Figure 5d and Figure S6) confirms this risk.

After contractions appear in cardiac tissues and trabeculae following dofetilide administration (Nguyen et al., 2017), as well as, in myocardial slices containing titin and collagen administered with isoproterenol (Watson et al., 2019). The EAD/DAD‐induced mechanical response predicted by our model (Figure 6) closely matches the experimental findings reported for hV‐CMs (Nguyen et al., 2017) and hiPSC‐CMs (Novak et al., 2012). This makes hiPSC‐CM‐CE suitable to be the baseline for population‐based studies aimed to assess drug cardiotoxic effects on contractility, in addition to electrophysiology.

### 4.3 Limitations and future works

While the model presented here captures some of the key electromechanical results of hiPSC‐CMs, studying the intrinsic heterogeneity of myofilaments, which may differ in different known morphologies of hiPSC‐CMs, needs further studies and simulations. Furthermore, the whole system has been implemented with ordinary differential equations (ODEs), i.e., the explicit considerations of spatial aspects have not been considered. To maintain an implementable system of ODEs, the CE model involves approximations. However, the approximations considered in Ca^2+^‐based myofilament activation and mean XB strains are used to fill the gap due to spatial scales, including local interactions, which are important but cannot be explicitly modeled by mean‐field approaches. The tension–SL relationship of the skinned version of CE was the only result that has been compared against the experimental data of cat trabeculae due to the lack of corresponding data for hiPSC‐CMs.

Although our inter‐scale analysis shows that the hiPSC‐CM‐CE model accurately predicts the inotropic effects of non‐cardiomyocytes in hiPSC‐CMs tissues, it is still a simplification of the actual system as accounting for the effect of heterogeneous hiPSC‐CMs substrates, and myosin expression in tissues requires a more comprehensive modeling framework.

There are other recent ionic models of hiPSC‐CMs (Kernik et al., 2019; Koivumäki et al., 2018) for which observing the effect of integrating the novel CE would be of interest, as the three models were already compared for their responses to drugs in Paci, Koivumäki, et al. (2021). However, we consider it out of the scope of this paper because of the following reasons. The intracellular compartmentalization of the Koivumäki2018 would make the CE integration extremely complex. Conversely, the Kernik2019 model shares the same compartmentalization of the Paci2020 model. However, the CE integration would still present specific challenges due to differences in the Ca^2+^ handling between the Paci2020 and the Kernik2019 models. The latter simulates a 2.5 times higher CaT peak and a 50% greater *I_CaL_* amplitude. Considering the non‐linearity of Ca^2+^ cooperation and sensitivity in the CE, a different reparameterization would be needed. Simply adding the current reparameterization of the Rice CE to the Kernik2019 model would just result in an electromechanical model whose simulated results are not consistent with experimental ranges.

## 5 CONCLUSIONS

Throughout the literature, the bulk of research in mathematical modeling for cardiomyocytes has been focused on electrophysiology. Specifically, excitation–contraction coupling in the myocardium or the association of the electrical and the mechanical parties has garnered less attention. hiPSC‐CMs are instrumental in developing patient‐specific models and cardiotoxicity tests at the cell level. Therefore, we have proposed a genuine electromechanical hiPSC‐CM model named hiPSC‐CM‐CE. Notably, fractional cell shortening, contraction RT_50_, the amplitude of tension, and the inotropic effect of non‐cardiomyocytes have been recapitulated within experimental ranges by the hiPSC‐CM‐CE. Lastly, the drug‐induced arrhythmogenic and inotropic effects and the aftercontractions due to triggered EADs have been simulated and validated against experimental data. The current model is a capable tool for extensions and translations of the findings toward *in silico* and *in vitro* in tissue‐ and organ‐level.

## CONFLICT OF INTEREST

The authors declare no conflict of interests.

## Supporting information



Supplementary Material
