# Supplementary material for: A mathematical model of hiPSC cardiomyocytes electromechanics
Source: Physiol Rep. 2021 Nov 25;9(22):e15124. doi: 10.14814/phy2.15124 (PMC8617339; doi:10.14814/phy2.15124)
Supplement: Supplementary file 1 — Supplementary Material [file PHY2-9-e15124-s001.docx]

# Supplementary Materials

# The hiPSC-CM-CE

Here, we give the new equations of the hiPSC-CM-CE model with the relevant equations from the original contractile element (Rice *et al.*, 2008). *SL*s denote sarcomere lengths at different conditions in µm, the $\left[ Troponin \right]$ is total troponin concentration in µM, and *s* is a variable indicating the sarcomere length.

# Constants

$${SL}_{rest}=1.9$$

$${SL}_{collagen}=2.25$$

${PCon}_{titin}=0.002$

$${PExp}_{titin}=10$$

$${PCon}_{collagen}=0.02$$

$${PExp}_{collagen}=70$$

$$\left[ Troponin \right]=70$$

# Flux of Ca2+ towards myofilament

$\left[ {Trop}_{Apr}Ca \right]=[Troponin]\times{Trop}_{Apr}(s)$ (S1)

$\frac{d}{dt}\left[ {Trop}_{Apr}Ca \right]=[Troponin]\times\frac{d}{dt}{Trop}_{Apr}(s)$ (S2)

# The Muscle Model

$\frac{d}{dt}SL=\frac{{Integral}_{Force}+({SL}_{0}-SL)\times vsc}{mass}$ (S3)

${Integral}_{Force}=\int_{0}^{t} (F_{active}\left( s \right)+F_{passive}\left( s \right)-F_{preload}-F_{afterload}(s))dt$ (S4)

$F_{afterload}(s)=0$ (S5)

$F_{preload}=\left\{ \begin{aligned} F_{passive}({SL}_{0}), &if {SL}_{0}\neq{SL}_{rest} \\ 0, &if {SL}_{0}={SL}_{rest} \end{aligned} \right.$ (S6)

# The Passive Force

$F_{titin}\left( x \right)=\left\{ \begin{aligned} -{PCon}_{titin}\times(\exp\left( {PExp}_{titin}\times\left( {SL}_{rest}-s \right) \right)-1), &s<{SL}_{rest} \\ {PCon}_{titin}\times(\exp\left( {PExp}_{titin}\times\left( s-{SL}_{rest} \right) \right)-1), &s\geq{SL}_{rest} \end{aligned} \right.$ (S7)

$F_{collagen}\left( x \right)=\left\{ \begin{aligned} 0, &s<{SL}_{collagen} \\ {PCon}_{collagen}\times(\exp\left( {PExp}_{collagen}\times\left( s-{SL}_{collagen} \right) \right)-1), &s\geq{SL}_{collagen} \end{aligned} \right.$ (S8)

# Supplementary Figures


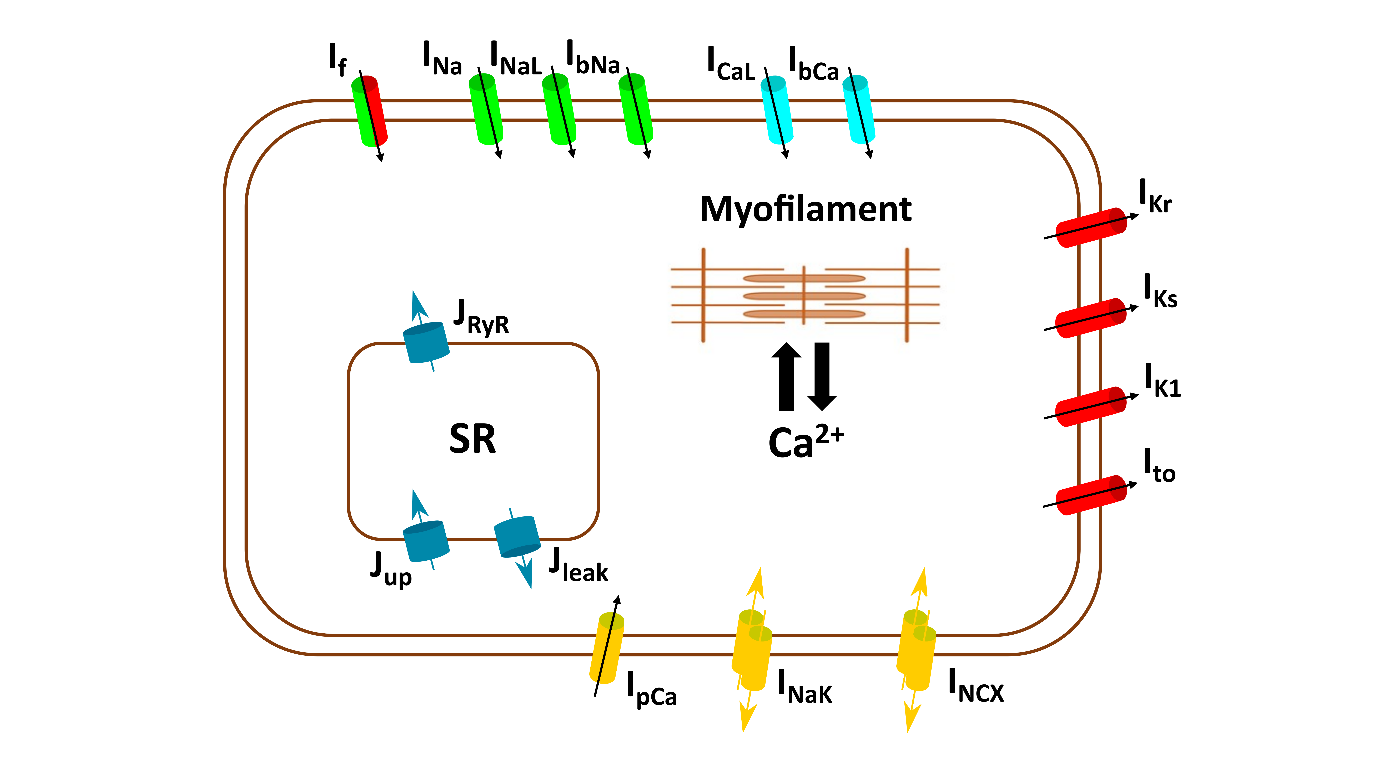


Fig. S1 A schematic diagram of the model showing cell compartments and main functional components. SR represents sarcoplasmic reticulum.


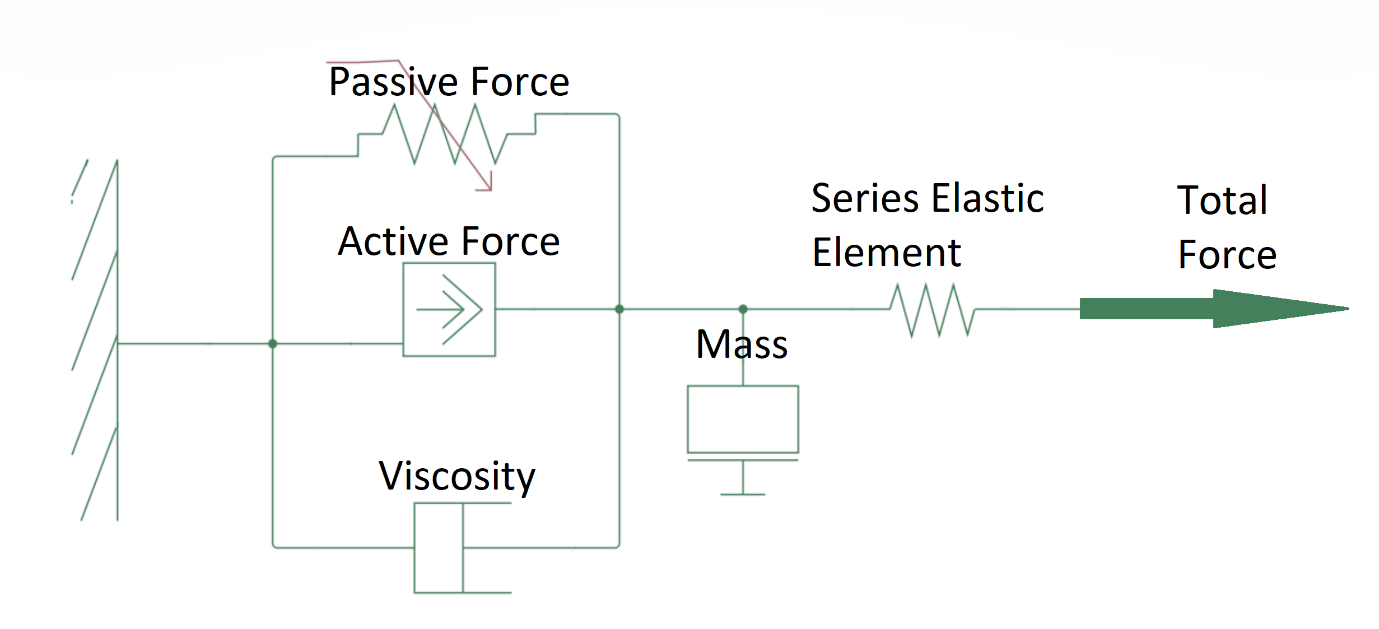


Fig. S2 The schematic representation of the contractile element used in the hiPSC-CM-CE model adapted from (Forouzandehmehr et al., 2020).


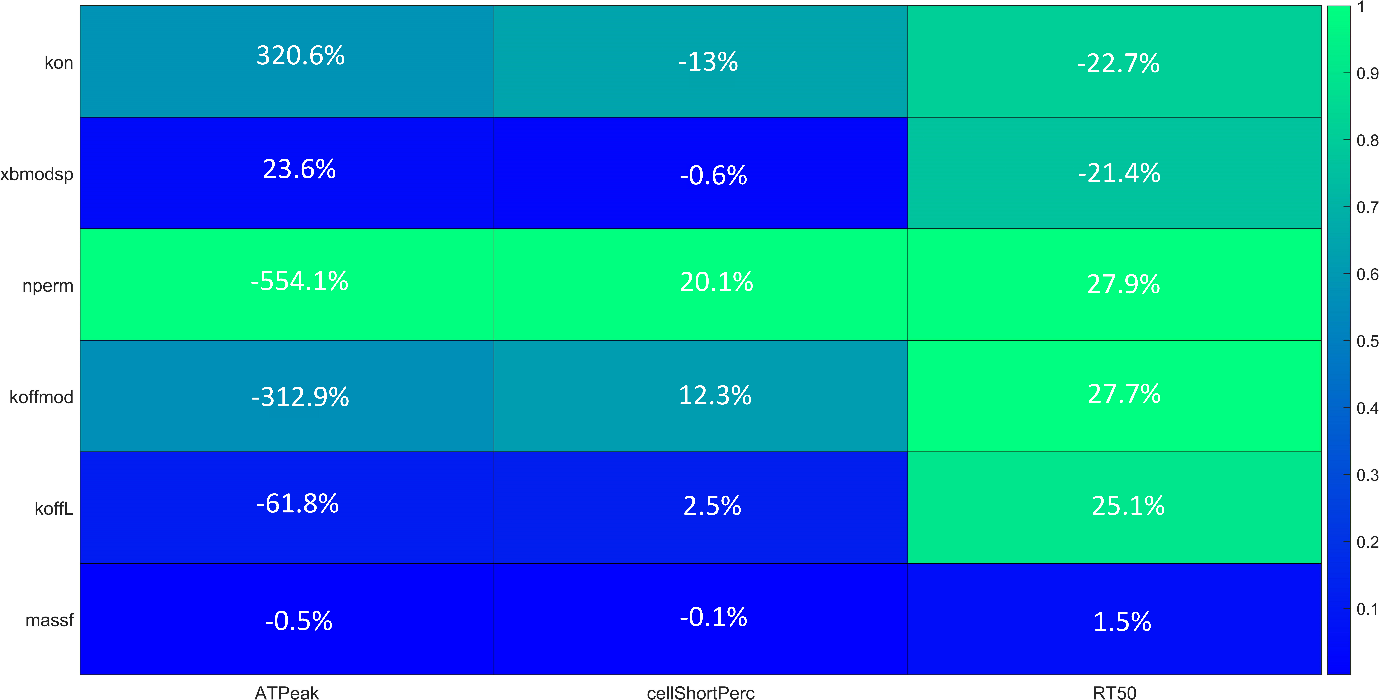


Fig. S3 The sensitivity analysis performed on hiPSC-CM-CE model. The parameter alterations were within ±15 of the calibrated values. The colour bar shows the relative sensitivity and the percents in each rectangle denote the maximum absolute sensitivity of the biomarker to the related parameter. ATpeak: Active Tension Peak, cellShortPerc: fractional cell shortening (%).


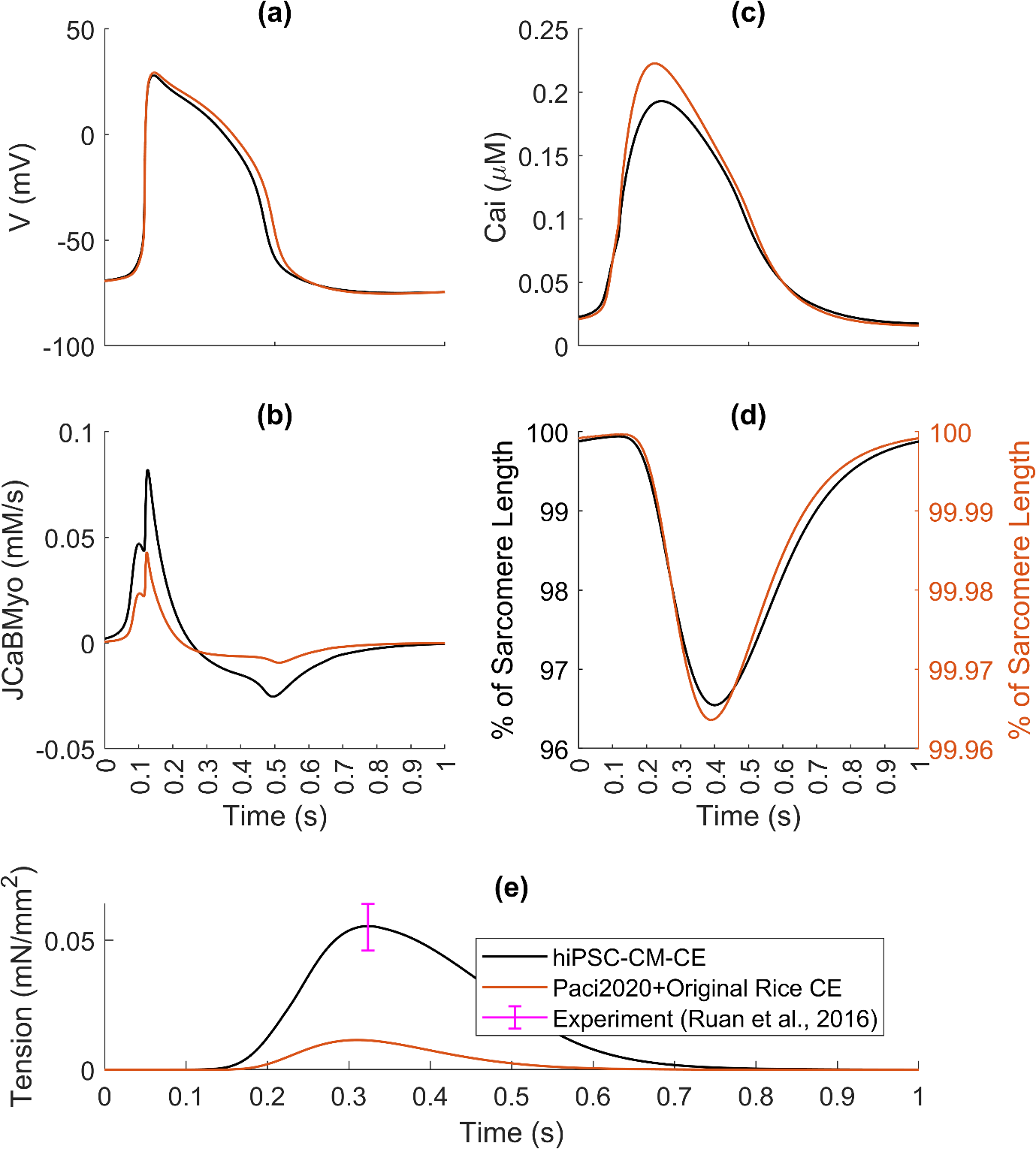


Fig. S4 Membrane potential (a), Ca^2+^ fluxes towards myofilament (b), CaTs (c), % of cell shortening (d), and active tensions (e) of the hiPSC-CM-CE model compared with the Paci2020+Original Rice CE.


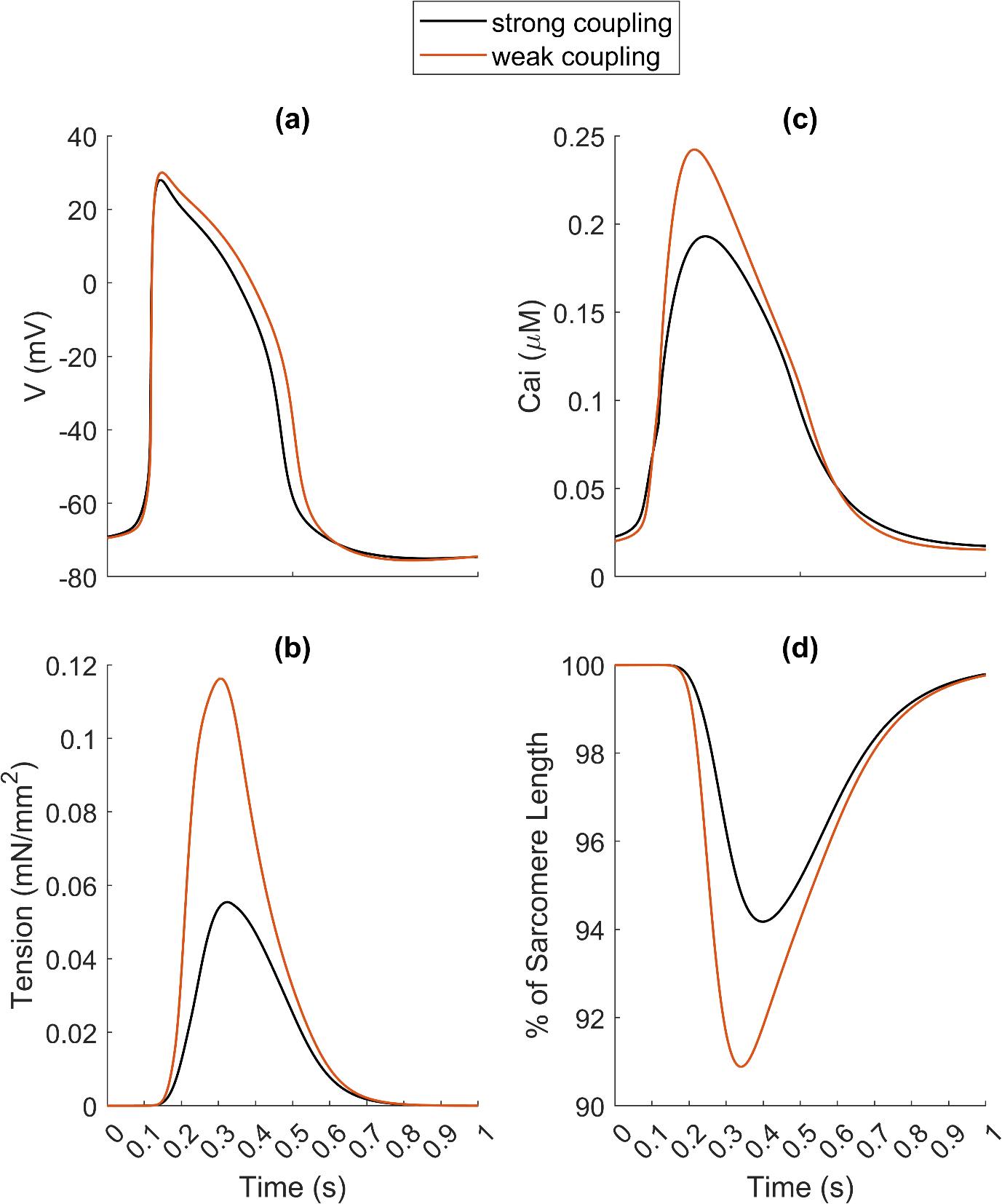


Fig. S5 hiPSC-CM-CE results in strong (with the myofilament feedback to the cytosolic Ca^2+^ dynamics) and weak coupling (no feedback). Membrane potential (a), Tension (b), Cytosolic Ca^2+^ concentration (c), and fractional cell shortening (d).


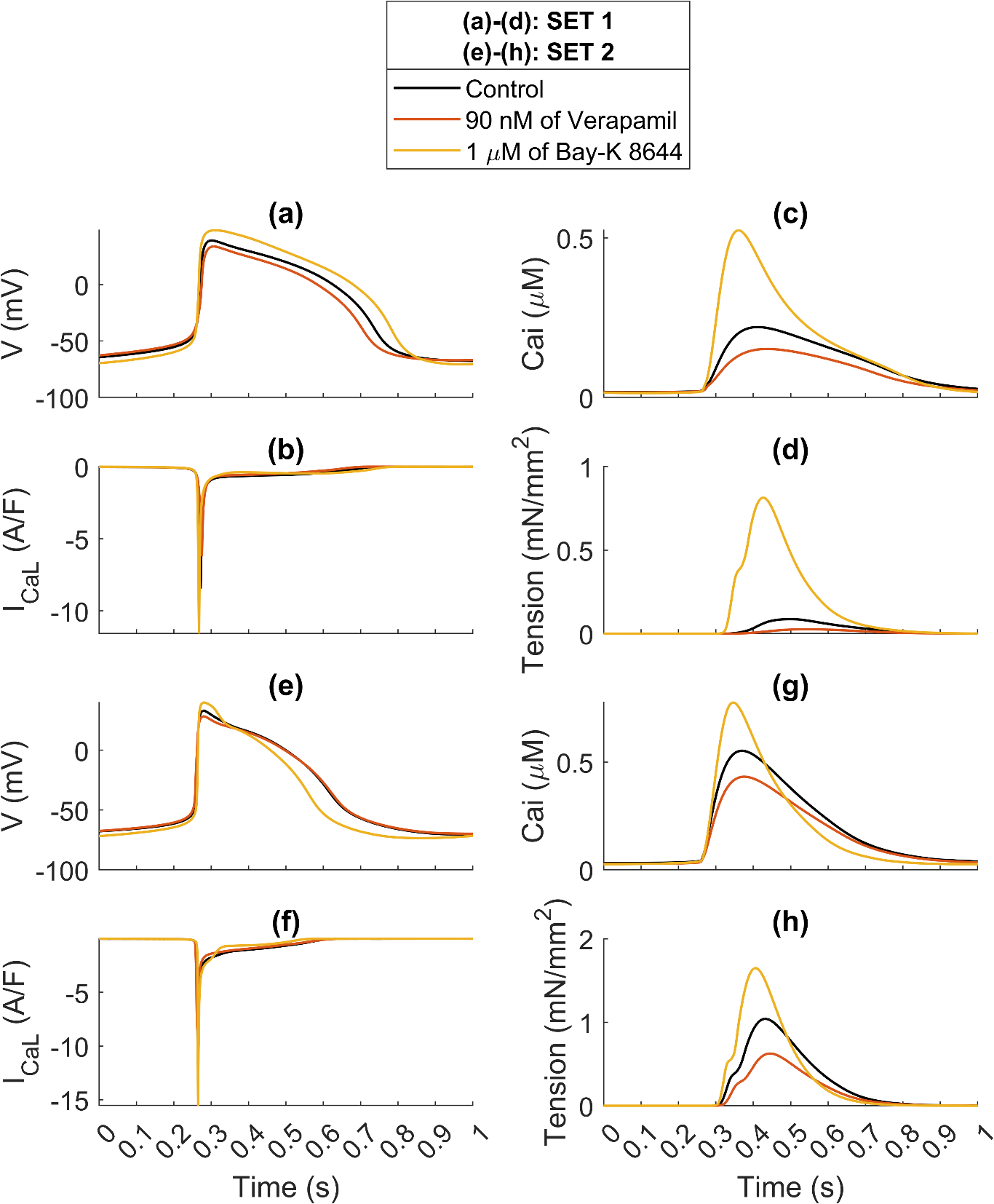


Fig. S6 Drug test results reproduced with the coefficient sets, SET1 and SET2, used to generate EADs. Notice that SET1 and SET2 correspond to the coefficients used to simulate Fig. 6 cases (a) and (b), respectively.


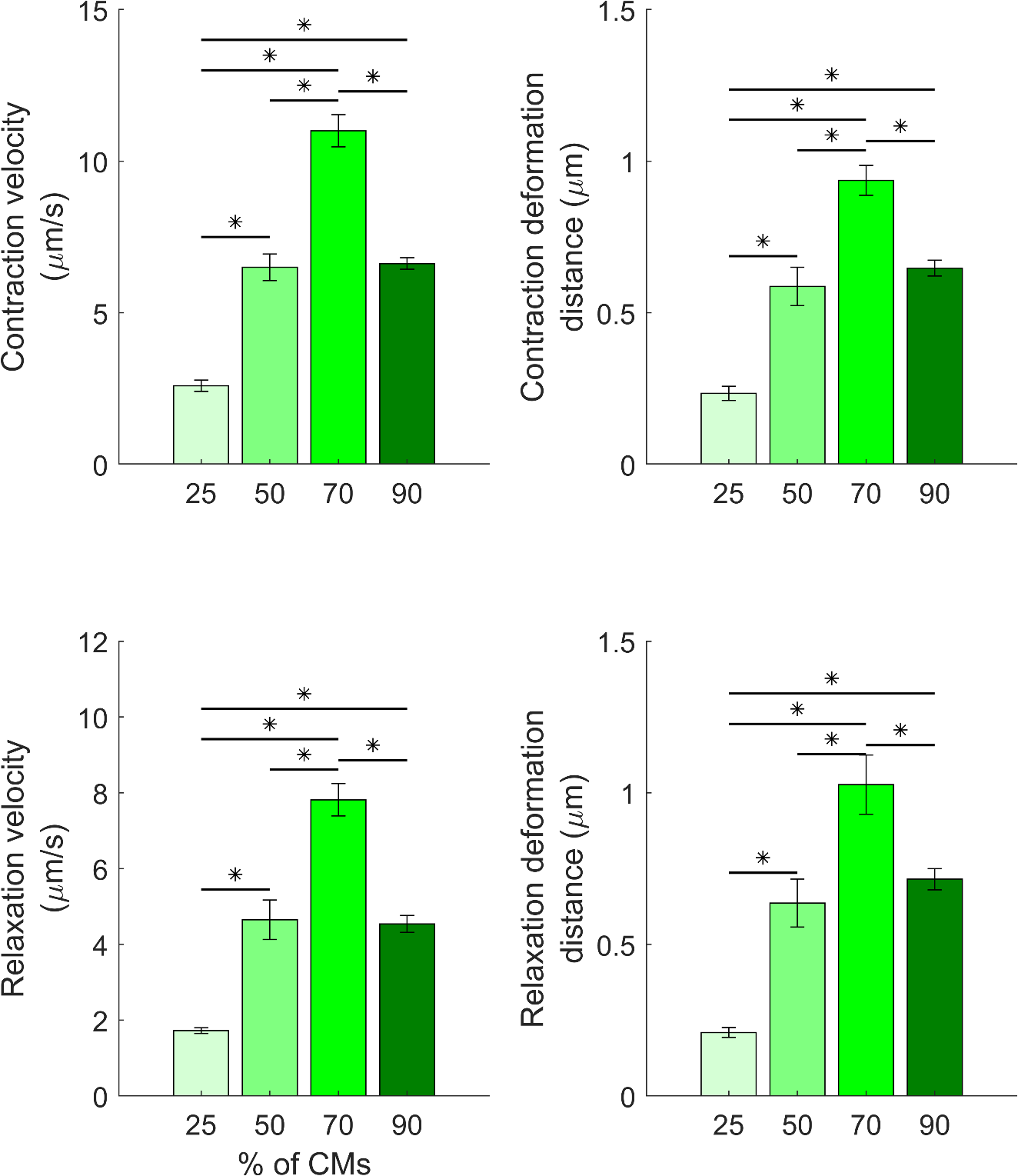


Fig. S7 Contractile characteristics of engineered heart tissues corresponding to different ratios of cardiomyocytes. These in vitro contraction-/relaxation-motion wave forms were obtained by cell-motion analysis. This figure was entirely redrawn using the experimental values presented in (Iseoka et al., 2018) (original Fig. 5). *p < 0.01.

# Supplementary Tables

Table S1 The IC_50_s and Hill coefficients used in simulations of 90 nM of Verapamil administration (Kramer et al., 2013). I_Na_ is fast Na^+^ current, I_Kr_ is rapid delayed rectifier K^+^ current, and I_CaL_ is L-type Ca^2+^ current.

| Item | I_Na_ | I_Kr_ | I_CaL_ |
| --- | --- | --- | --- |
| IC_50_ | 32.5 | 0.25 | 0.2 |
| Hill coef. | 1.33 | 0.89 | 0.8 |


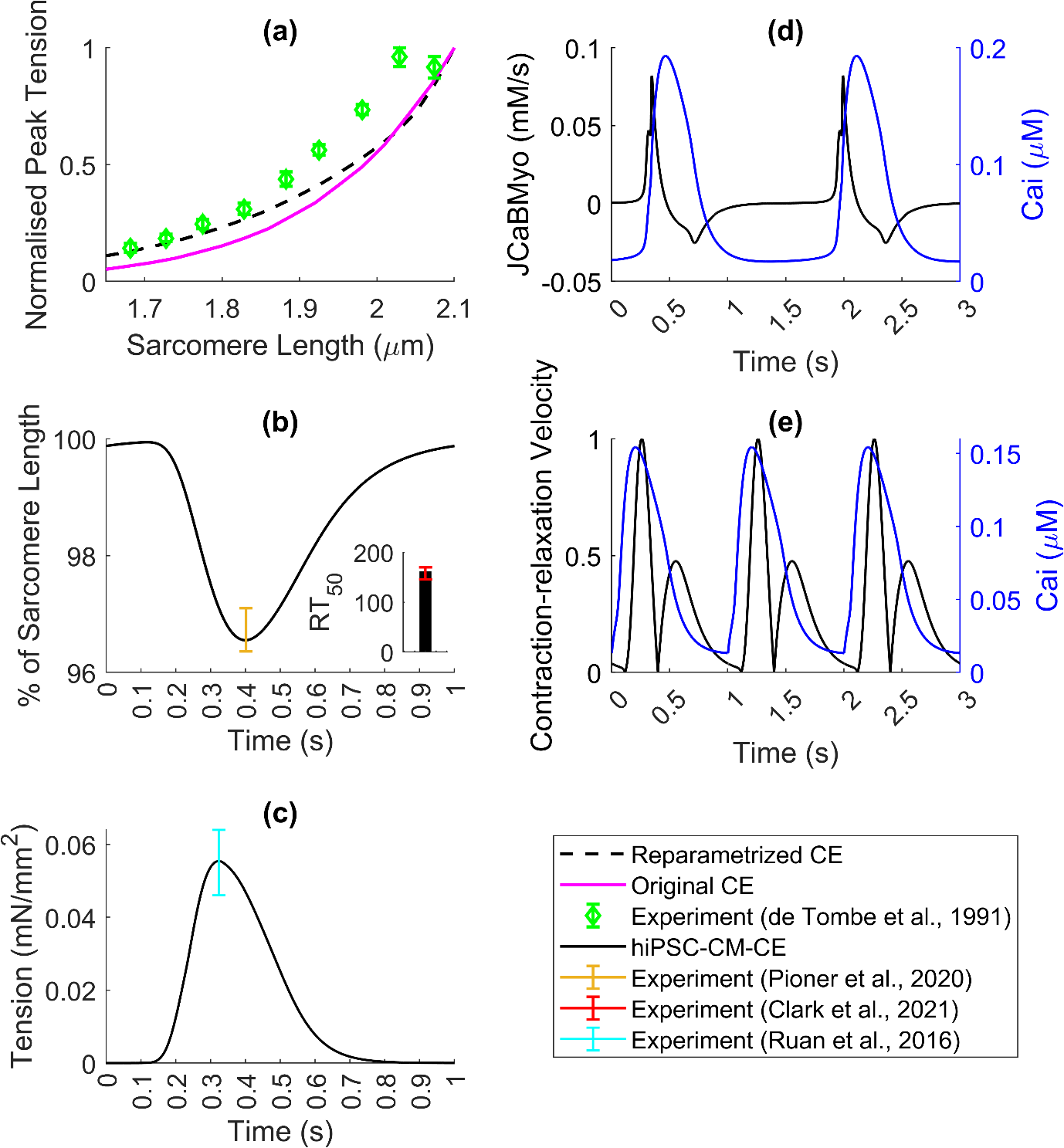


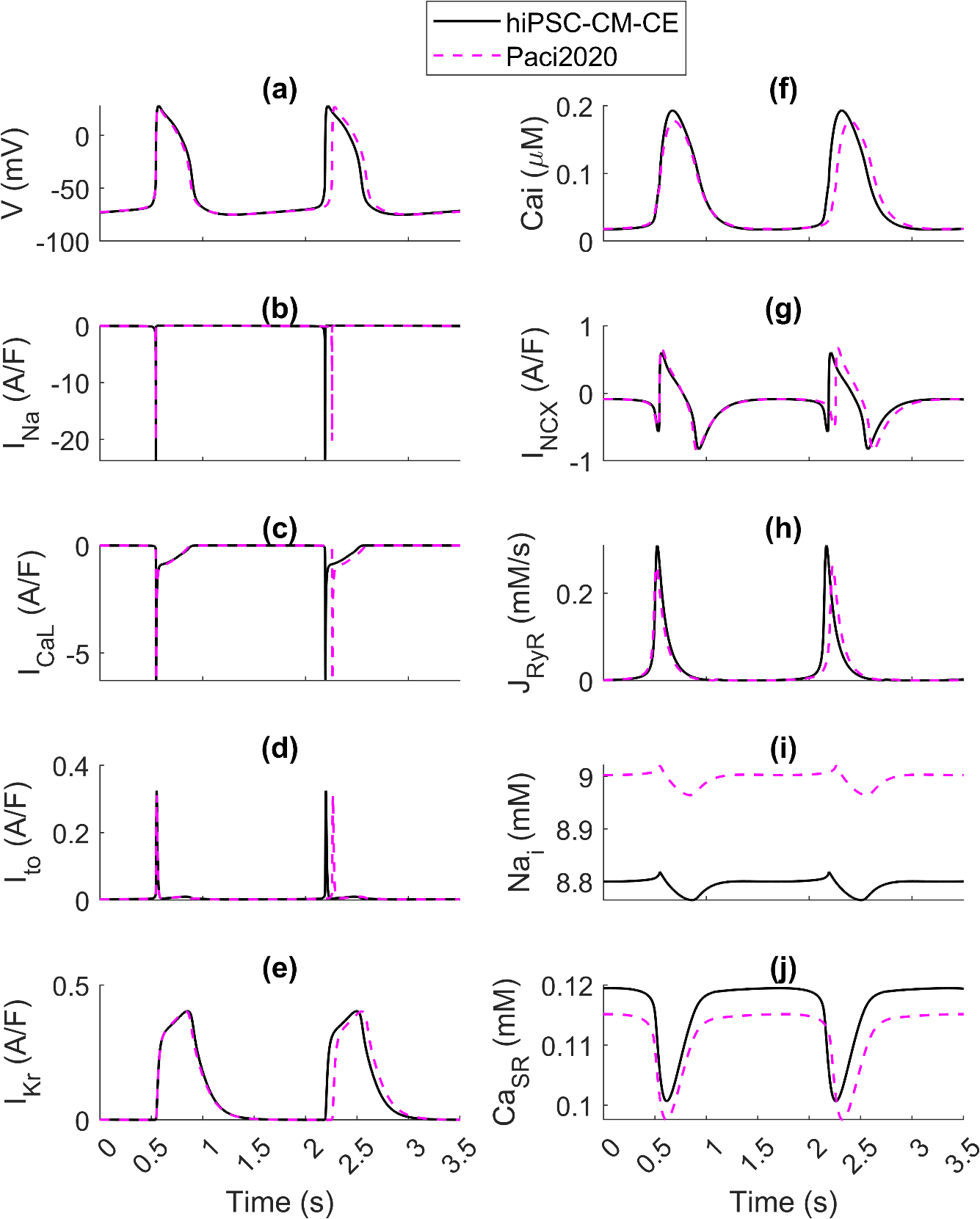


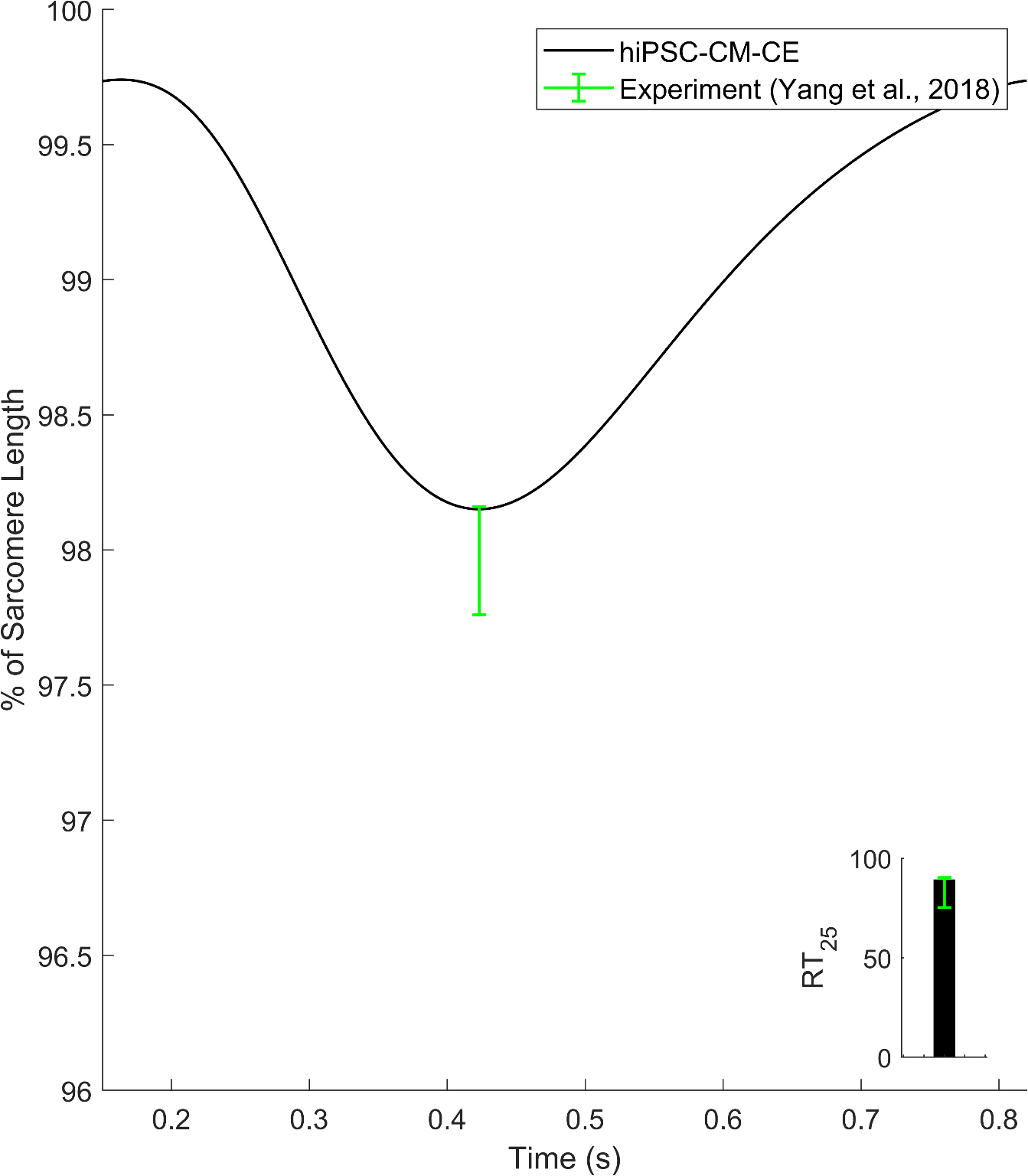


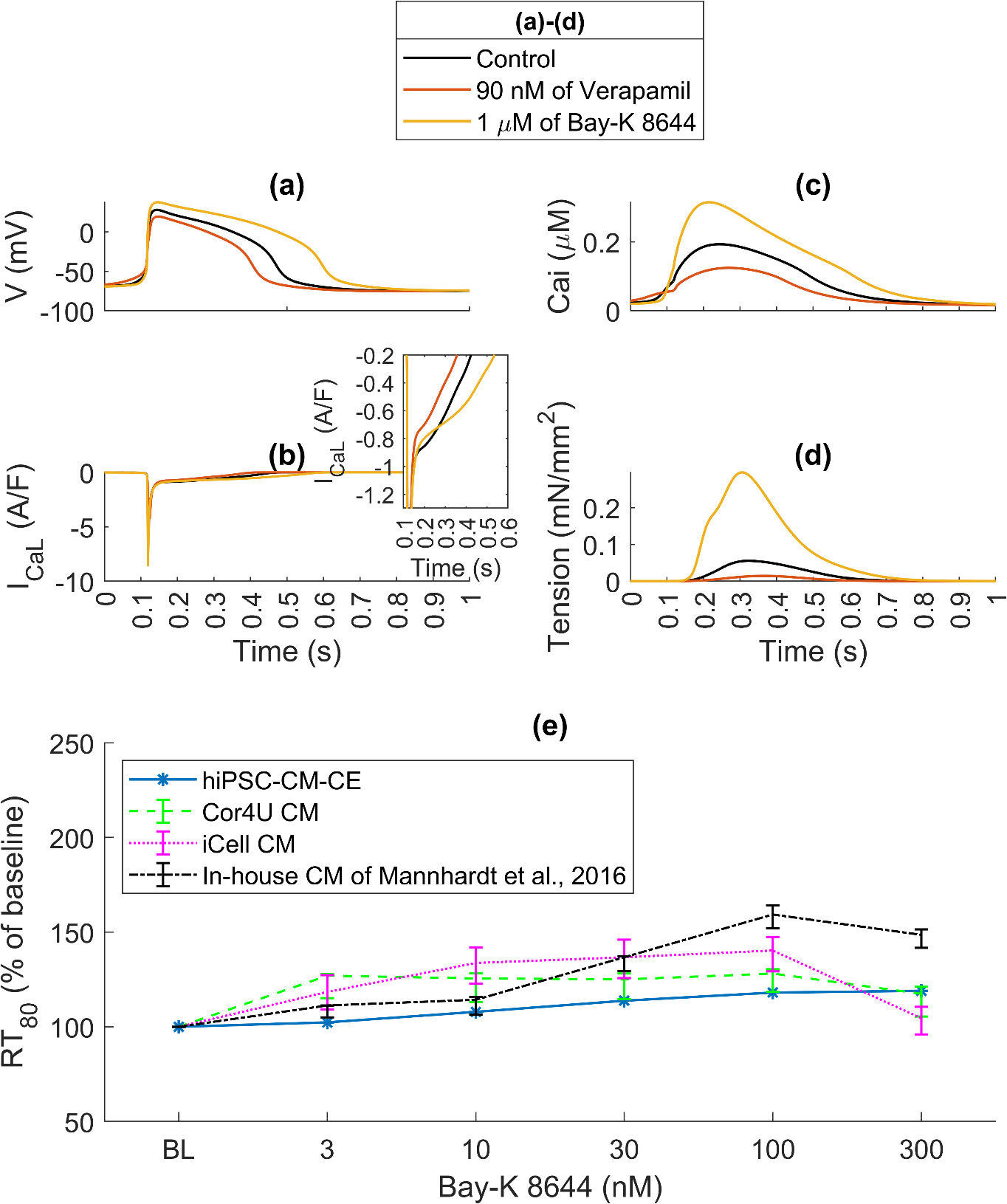


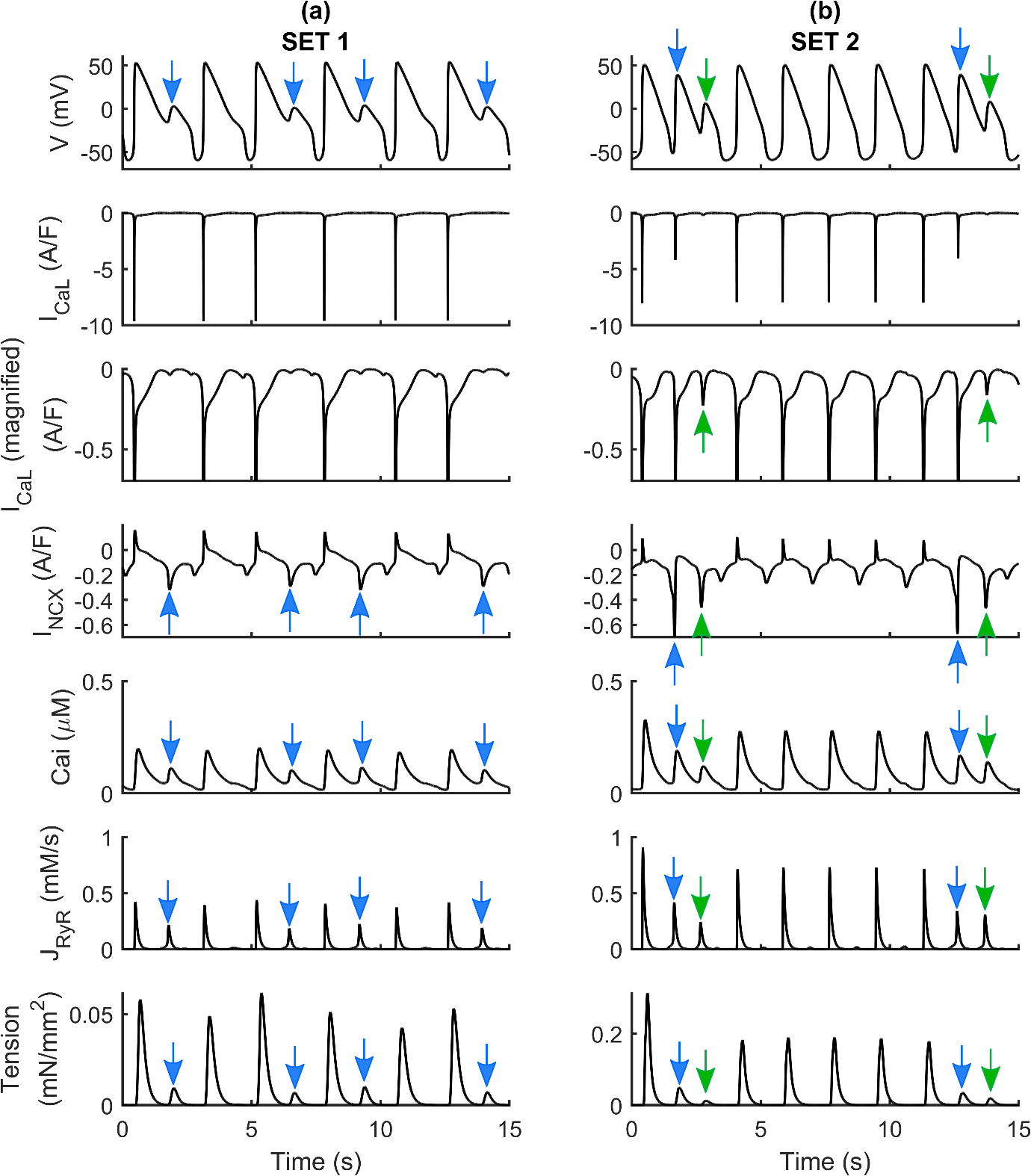


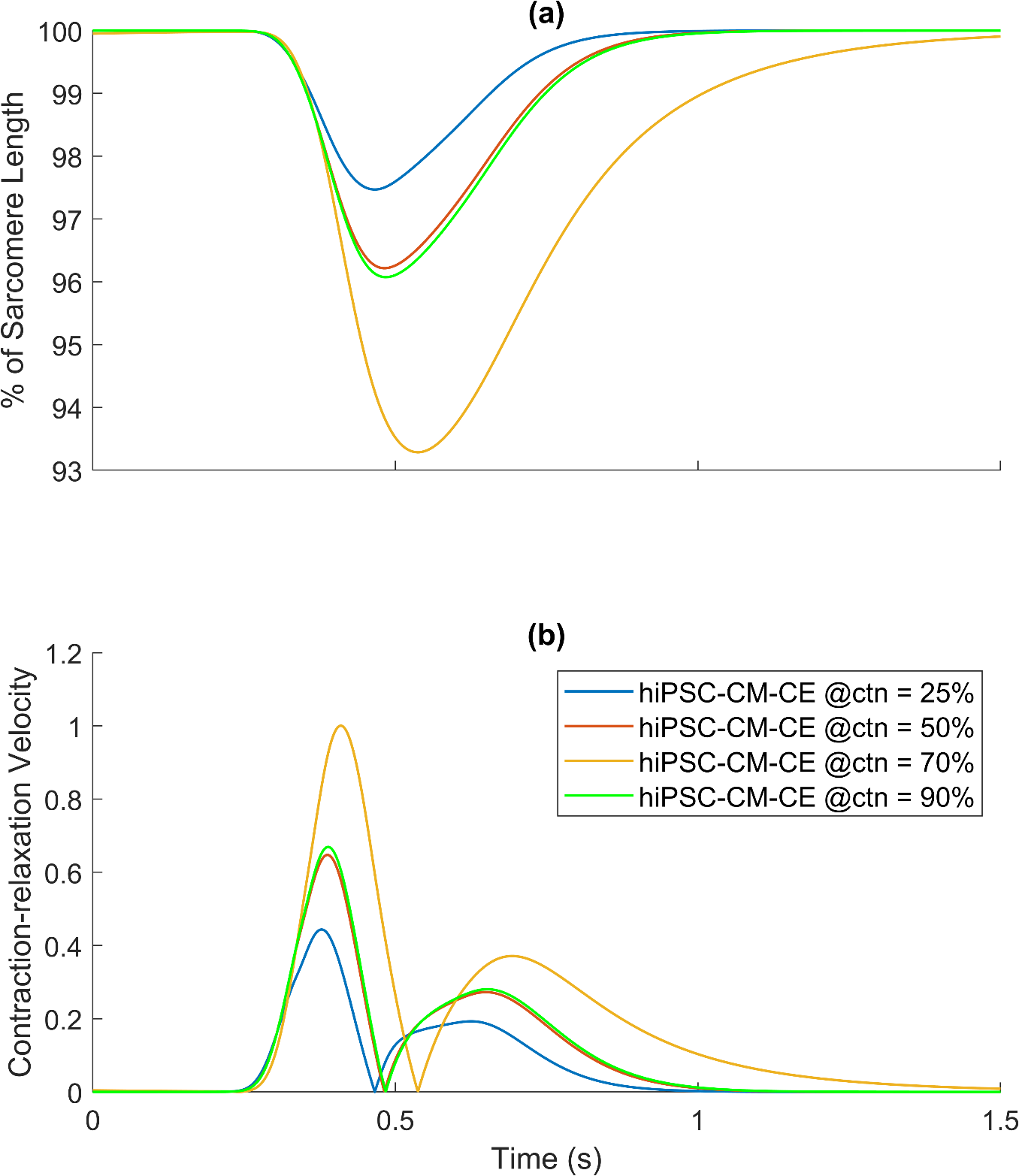


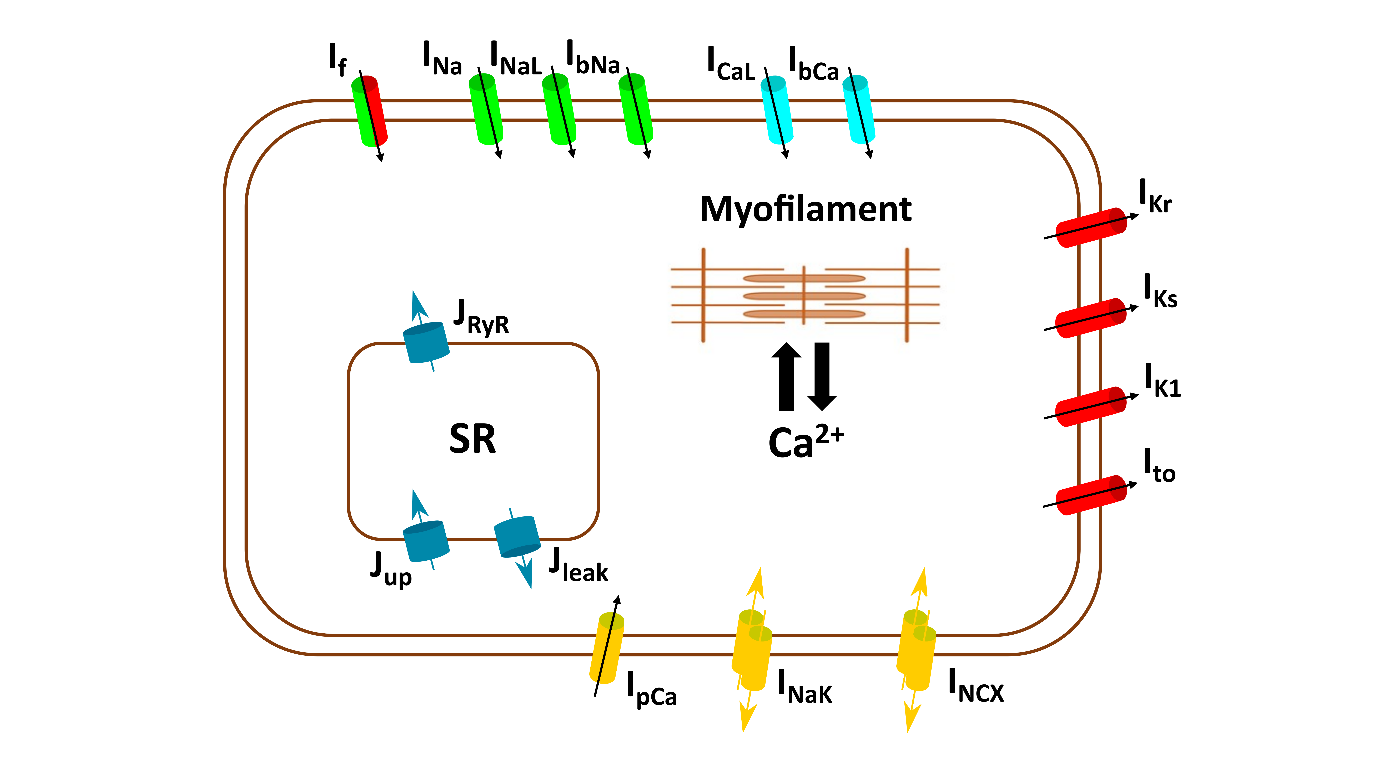


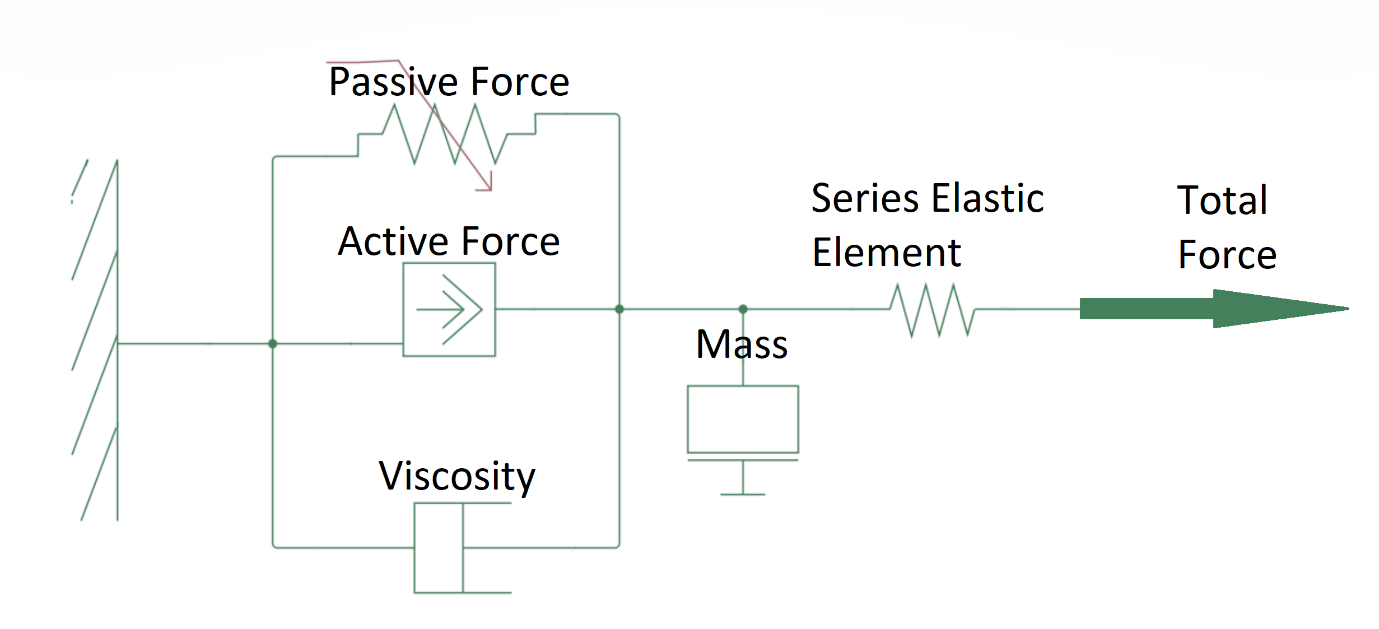


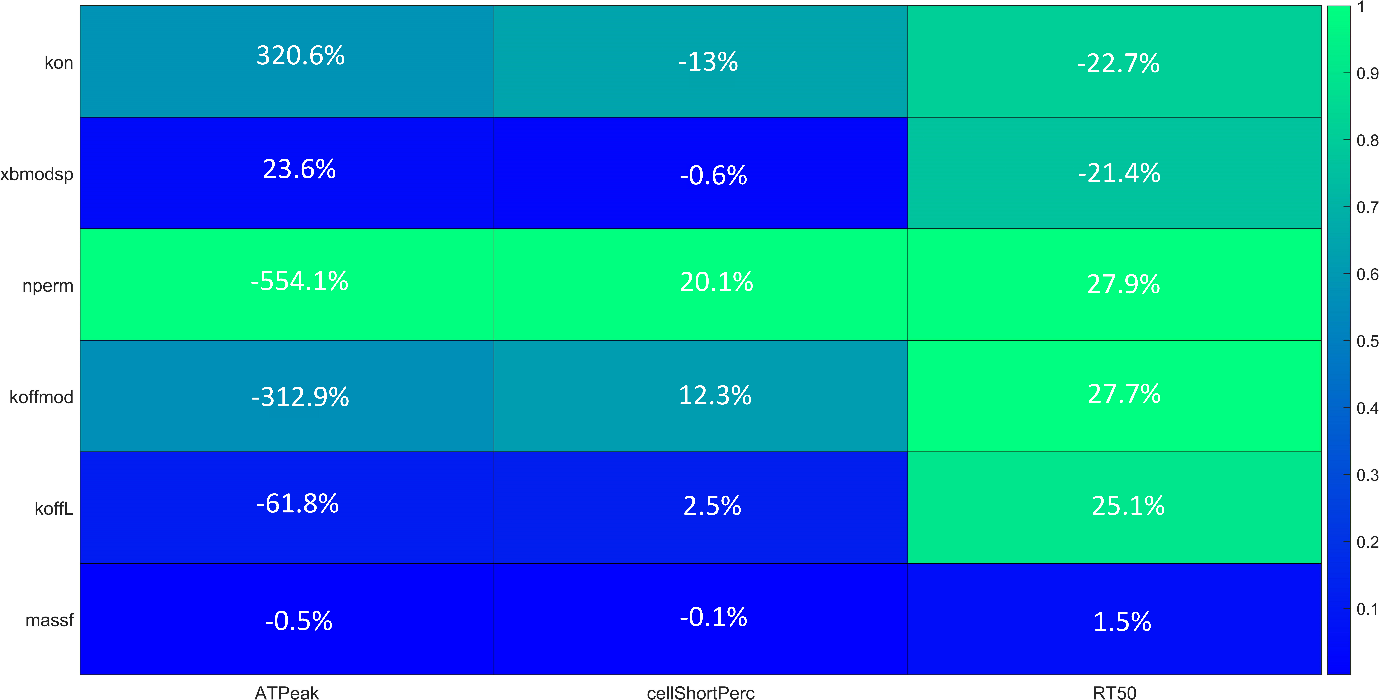


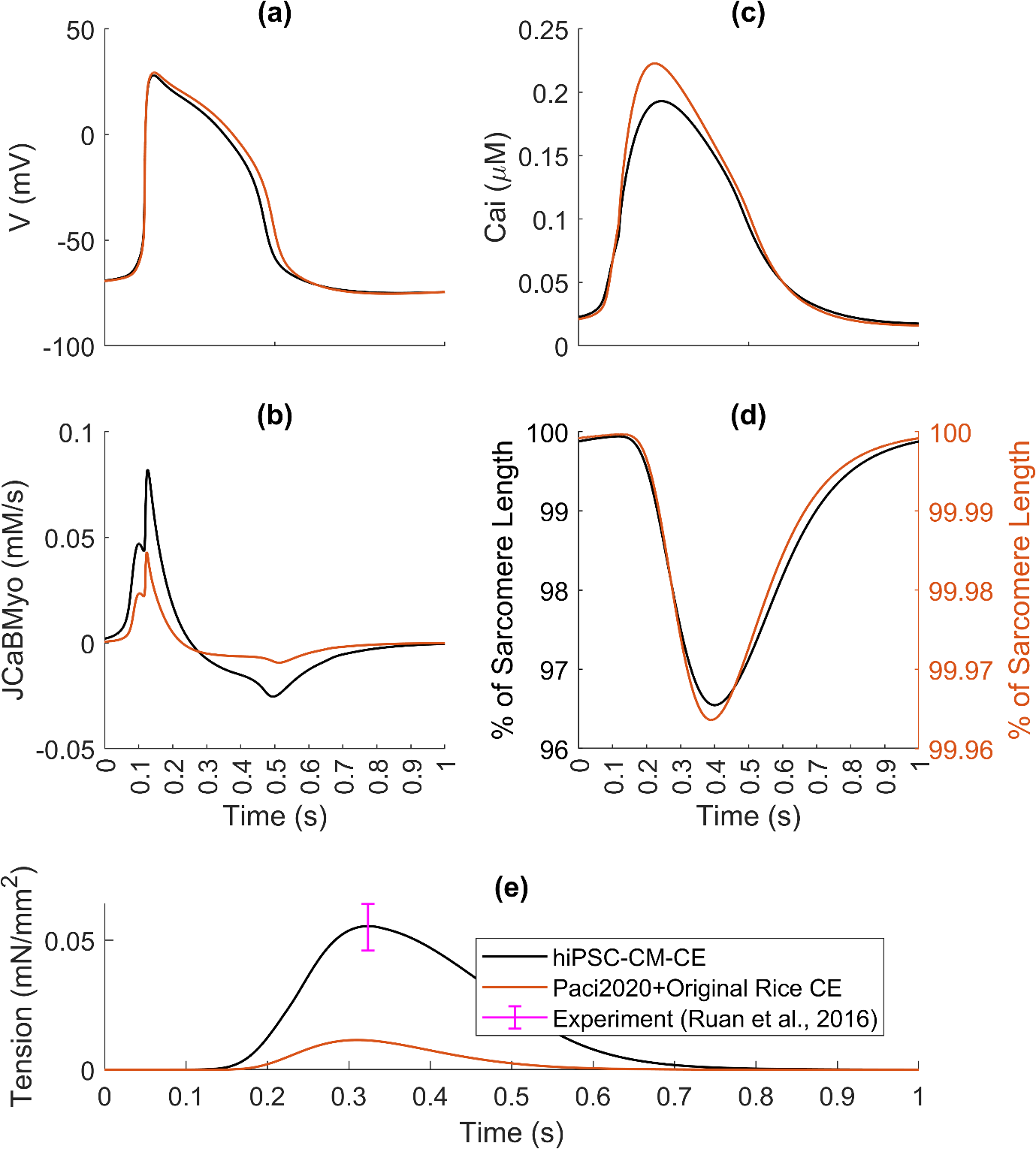


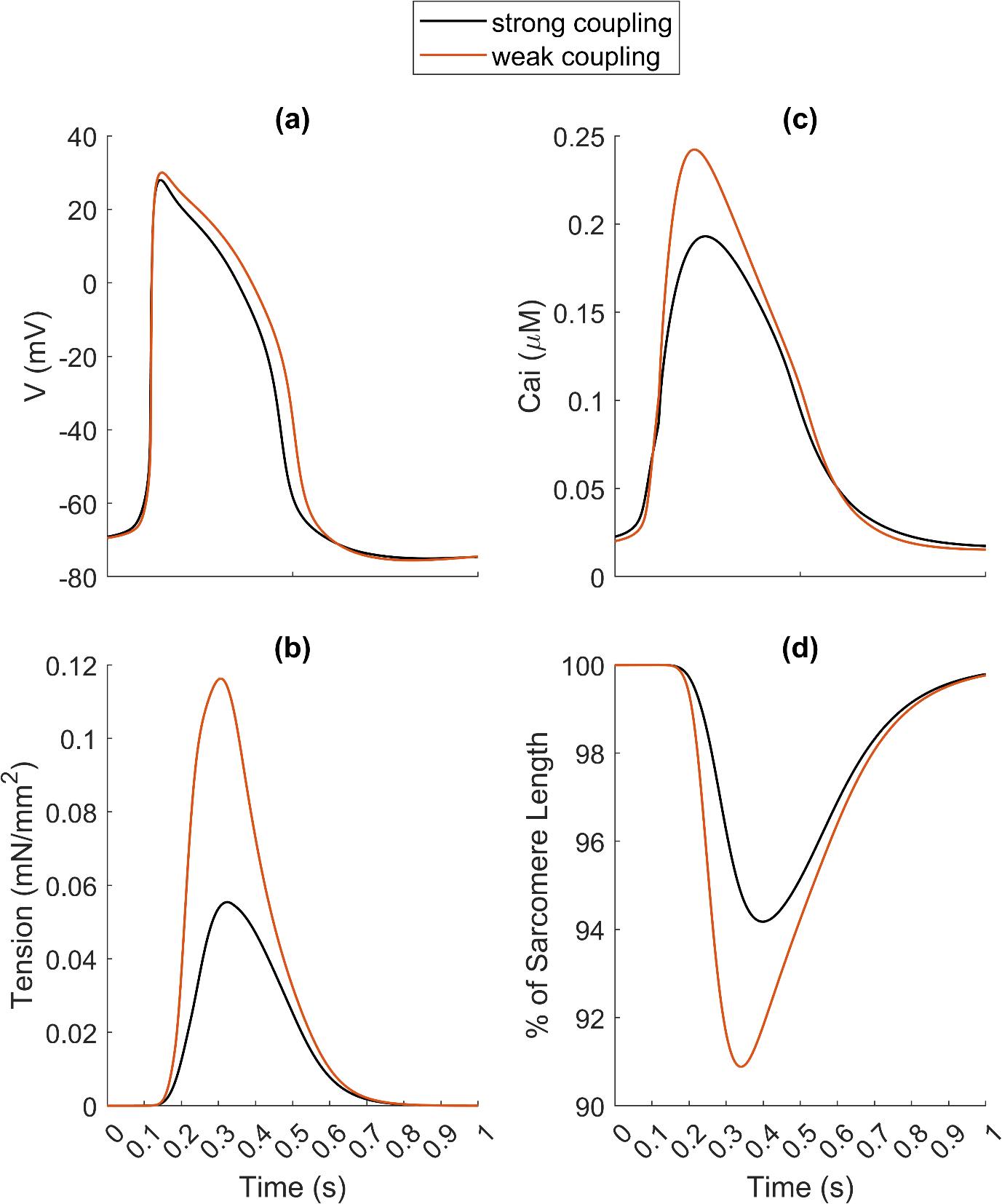


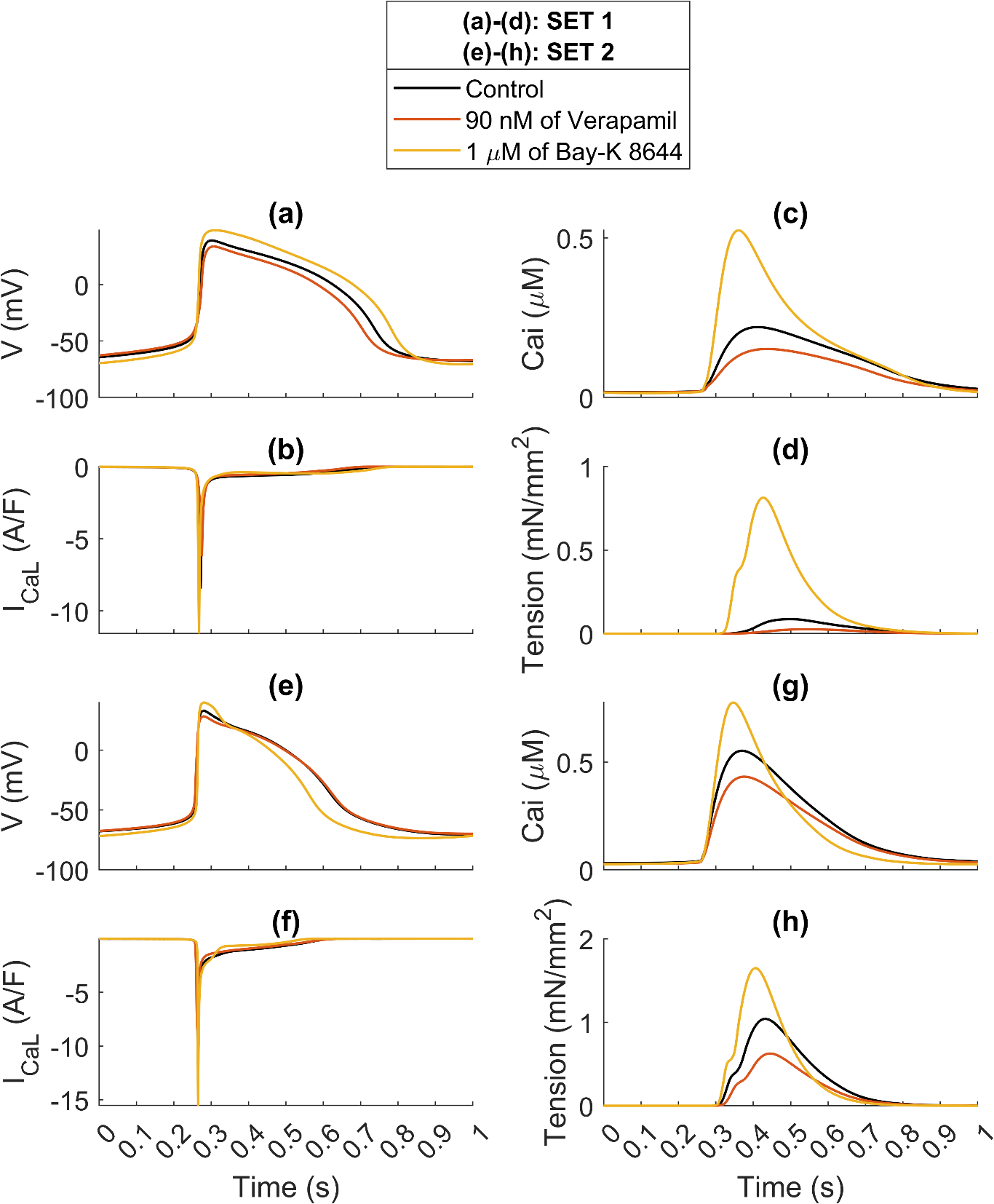


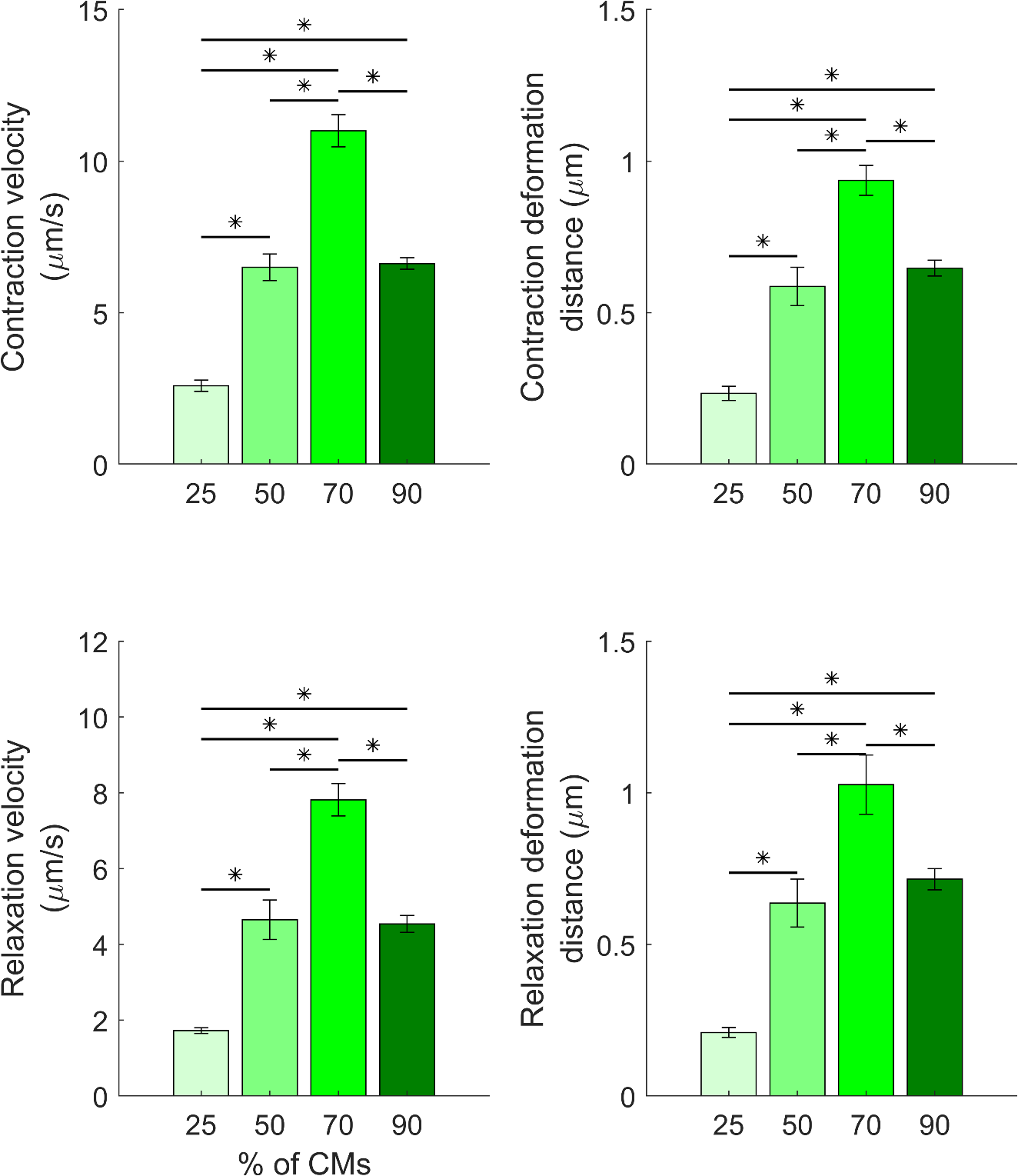


| **Parameter** | **Baseline Value** | **The hiPSC-CM-CE** | **% of change** |
| --- | --- | --- | --- |
| **K_on_ (s^-1^ mM^-1^)** | 50×10^3^ | 62.5×10^3^ | 25 |
| **K_offL_ (s^-1^)** | 250 | 200 | -20 |
| **K_offH_ (s^-1^)** | 25 | 25 | 0 |
| **perm_50_** | 0.5 | 0.6 | 20 |
| **n_perm_** | 15 | 11.28 | -24.8 |
| **K_n-p_ (s^-1^)** | 500 | 550 | 10 |
| **K_p-n_ (s^-1^)** | 50 | 50 | 0 |
| **K_offmod_** | 1 | 0.5 | -50 |
| **m (s^2^ µm^-1^)** | 5×10^-5^ | 2×10^-5^ | -60 |
| **kxb** | 120 | 12 | - |
| **xbmodsp** | 1.33,1,0.2 | 0.2 | - |

| **#** | **Experimental Paper** | **[Ca^2+^]_e_** | **Temperature (**$\boldsymbol{℃}$**)** | **Biomarkers used for the reparameterization** |
| --- | --- | --- | --- | --- |
| **1** | Pioner et al., 2020 (Pioner *et al.*, 2020) | 1.8 mM | 37 | % of Cell shortening, |
| **2** | Clarks et al., 2021 (Clark *et al.*, 2021) | 1.8 mM | 37 | Contraction RT_50_ |
| **3** | Yang et al., 2018 (Yang *et al.*, 2018) | 1.8 mM | 37 | % of Cell shortening, Contraction RT_25_, |
| **4** | Ruan et al., 2016 (Ruan *et al.*, 2016) | 1.8 mM | Mechanical Measurements and drug tests at 37, Histological Measurements and microscopy at room temp. | Active Tension Amplitude and Inotropic effects of Verapamil and Bay-K 8644 |
| **5** | Iseoka et al., 2018 (Iseoka *et al.*, 2018) | N/A | 37 | Inotropic effects of non-cardiomyocytes on the mechanical outputs of hiPSC-CMs |
| **6** | Hayakawa et al., 2014 (Hayakawa *et al.*, 2014) | N/A | 37 | Contraction Velocity Profile |
| **7** | Rodriguez et al., 2014 (Rodriguez *et al.*, 2014) | N/A | 37 | Contraction Velocity Profile |

| No. | Biomarker | Paci2020 | Paci2020+Original Rice CE | **hiPSC-CM-CE** | Exp. Value (Mean±SD) |
| --- | --- | --- | --- | --- | --- |
| 1 | APA (mV) | 102 | 105 | **103** | 104±6 |
| 2 | MDP (mV) | -74.9 | -75.3 | **-75.0** | -75.6±6.6 |
| 3 | AP CL (ms) | 1712 | 1559 | **1644** | 1700±548 |
| 4 | dV/dt max (V/s) | 20.5 | 14.0 | **23.9** | 27.8±26.3 |
| 5 | APD_10_ (ms) | 87.0 | *109.5* | **95.0** | 74.1±26.3 |
| 6 | APD_30_ (ms) | 224 | *259* | **238** | 180±59 |
| 7 | APD_90_ (ms) | 390 | 421 | **403** | 415±119 |
| 8 | AP Tri | 2.8 | 3.2 | **2.9** | 2.5±1.1 |
| 9 | CaT DURATION (ms) | 691 | 681 | **693** | 805±188 |
| 10 | CaT tRise_10, peak_ (ms) | 184 | *136* | **163** | 270±108 |
| 11 | Cat tRise_10,50_ (ms) | 54.9 | 39.2 | **46.2** | 82.9±50.5 |
| 12 | CaT tRise_10,90_ (ms) | 118 | *86* | **102** | 167±70 |
| 13 | CaT tDecay_90,10_ (ms) | 341 | 349 | **343** | 410±100 |

| Item | I_Na_ | I_Kr_ | I_CaL_ |
| --- | --- | --- | --- |
| IC_50_ | 32.5 | 0.25 | 0.2 |
| Hill coef. | 1.33 | 0.89 | 0.8 |

Fig. 1 Tuning parameters of the CE and their effect on the hiPSC-CM-CE. The green and black texts highlight the positive and negative effects on the mechanical outputs selected for validation, respectively.

Fig. 2 Mechanical biomarkers simulated by the hiPSC-CM-CE model. (a) Normalised peak tensions vs SL. (b) % of cell shortening and contraction RT_50_ (time from peak contraction to 50% relaxation) at 1Hz pacing. (c) The simulated tension profile at spontaneous beating. (d) The binding flux of Ca^2+^ towards the myofilament (JCaBMyo) and the CaT. (e) Normalised motion waveform (contraction-relaxation velocity) and the CaT at 1Hz pacing.

Fig. 3 Simulated action potentials and ionic currents of the hiPSC-CM-CE model vs Paci2020 (Paci et al., 2020) in spontaneous beating in the steady state condition. (a) membrane potential. (b) Fast Na^+^ current (I_Na_). (c) L-type Ca^2+^ current (I_CaL_). (d) Transient outward K^+^ current (I_to_). (e) Rapid delayed rectifier K^+^ current (I_Kr_). (f) Cytosolic Ca^2+^ concentration (Ca_i_). (g) Na^+^/Ca^2+^ exchanger (I_NCX_). (h) Ca^2+^ release from sarcoplasmic reticulum (J_RyR_). (i) Cytosolic Na^+^ concentration (Na_i_). (j) Sarcoplasmic Ca^2+^ concentration (Ca_SR_).

Fig. 4 Percent of cell shortening and the contraction RT_25_ (time from peak contraction to 50% of relaxation) simulated by the hiPSC-CM-CE model.

Fig. 5 The electrophysiology and contractility of the hiPSC-CM-CE in control and drug modes. (a) Action potentials. (b) L-type Ca^2+^ currents. (c) Cytosolic Ca^2+^ transients. (d) Active tensions. (e) RT_80_ (time from peak contraction to 80% of relaxation) results of the model in response to different concentrations of Bay-K 8644 and the in vitro data obtained from different hiPSC-CMs. Cor4U and iCell are commercial cardiomyocytes the data of which have been acquired from (Mannhardt et al., 2016). Cases (a-d) show data at spontaneous condition, and case (e) shows the model results at 1.5 Hz pacing. BL: Baseline. Of note, the same tests presented in panels (a)-(d) were also performed in paced conditions (1Hz), without observing noteworthy differences with the results presented in this figure.

Fig. 6 The action potentials, L-type Ca^2+^ currents (I_CaL_), Na^+^/Ca^2+^ exchangers (I_NCX_), Calcium Transients, Ca^2+^ releases from the sarcoplasmic reticulum (J_RyR_), and active tensions simulated for two sets of parameters (SET1 and SET2) used to generate models which develop EADs (A) and (B), using hiPSC-CM-CE as baseline. Blue arrows show almost full anticipated APs due to the strong first inward I_NCX_ activation. Then, a second I_NCX_ activation triggers the second EADs where we observe an I_CaL_ reactivation up to -0.22 pA/pF (green arrows). The scales of tensions are different. In the third line, we show a magnification of the I_CaL_ traces, highlighting I_CaL_ reactivation.

Fig. 7 Simulated percent of cell shortenings (a) and (b) contraction-relaxation velocities at different percents of cardiomyocytes in the engineered heart tissue (ctns), normalised over the maximum value simulated for ctn = 70%.

Fig. S1 A schematic diagram of the model showing cell compartments and main functional components. SR represents sarcoplasmic reticulum.

Fig. S2 The schematic representation of the contractile element used in the hiPSC-CM-CE model adapted from (Forouzandehmehr et al., 2020).

Fig. S3 The sensitivity analysis performed on hiPSC-CM-CE model. The parameter alterations were within ±15 of the calibrated values. The colour bar shows the relative sensitivity and the percents in each rectangle denote the maximum absolute sensitivity of the biomarker to the related parameter. ATpeak: Active Tension Peak, cellShortPerc: fractional cell shortening (%).

Fig. S4 Membrane potential (a), Ca^2+^ fluxes towards myofilament (b), CaTs (c), % of cell shortening (d), and active tensions (e) of the hiPSC-CM-CE model compared with the Paci2020+Original Rice CE.

Fig. S5 hiPSC-CM-CE results in strong (with the myofilament feedback to the cytosolic Ca^2+^ dynamics) and weak coupling (no feedback). Membrane potential (a), Tension (b), Cytosolic Ca^2+^ concentration (c), and fractional cell shortening (d).

Fig. S6 Drug test results reproduced with the coefficient sets, SET1 and SET2, used to generate EADs. Notice that SET1 and SET2 correspond to the coefficients used to simulate Fig. 6 cases (a) and (b), respectively.

Fig. S7 Contractile characteristics of engineered heart tissues corresponding to different ratios of cardiomyocytes. These in vitro contraction-/relaxation-motion wave forms were obtained by cell-motion analysis. This figure was entirely redrawn using the experimental values presented in (Iseoka et al., 2018) (original Fig. 5). *p < 0.01.

Table S1 The IC_50_s and Hill coefficients used in simulations of 90 nM of Verapamil administration (Kramer et al., 2013). I_Na_ is fast Na^+^ current, I_Kr_ is rapid delayed rectifier K^+^ current, and I_CaL_ is L-type Ca^2+^ current.
